# Differential expression of retinal determination genes in the principal and secondary eyes of *Cupiennius salei* Keyserling (1877)

**DOI:** 10.1186/s13227-015-0010-x

**Published:** 2015-04-28

**Authors:** Leyli Samadi, Axel Schmid, Bo Joakim Eriksson

**Affiliations:** Department of Neurobiology, Centre for Organismal Systems Biology, Faculty of Life Sciences, University of Vienna, Althanstraße 14, 1090 Vienna, Austria

**Keywords:** Eye development, Principal eyes, Retinal determination gene network, Secondary eyes, Spider

## Abstract

**Background:**

Transcription factors that determine retinal development seem to be conserved in different phyla throughout the animal kingdom. In most representatives, however, only a few of the involved transcription factors have been sampled and many animal groups remain understudied. In order to fill in the gaps for the chelicerate group of arthropods, we tested the expression pattern of the candidate genes involved in the eye development in the embryo of the wandering spider *Cupiennius salei*. One main objective was to profile the molecular development of the eyes and to search for possible variation among eye subtype differentiation. A second aim was to form a basis for comparative studies in order to elucidate evolutionary pathways in eye development.

**Results:**

We screened the spider embryonic transcriptome for retina determination gene candidates and discovered that all except one of the retinal determination genes have been duplicated. Gene expression analysis shows that the two orthologs of all the genes have different expression patterns. The genes are mainly expressed in the developing optic neuropiles of the eyes (lateral furrow, mushroom body, arcuate body) in earlier stages of development (160 to 220 h after egg laying). Later in development (180 to 280 h after egg laying), there is differential expression of the genes in disparate eye vesicles; for example, *Cs-otxa* is expressed only in posterior-lateral eye vesicles, *Cs-otxb*, *Cs-six1a*, and *Cs-six3b* in all three secondary eye vesicles, *Cs-pax6a* only in principal eye vesicles, *Cs-six1b* in posterior-median, and posterior-lateral eye vesicles, and *Cs-six3a* in lateral and principal eye vesicles.

**Conclusions:**

Principle eye development shows *pax6a* (*ey*) expression, suggesting *pax6* dependence, although secondary eyes develop independently of *pax6* genes and show differential expression of several retinal determination genes. Comparing this with the other arthropods suggests that *pax6*-dependent median eye development is a ground pattern of eye development in this group and that the ocelli of insects, the median eyes of chelicerates, and nauplius eyes can be homologised. The expression pattern of the investigated genes makes it possible to distinguish between secondary eyes and principal eyes. Differences of gene expression among the different lateral eyes indicate disparate function combined with genetic drift.

**Electronic supplementary material:**

The online version of this article (doi:10.1186/s13227-015-0010-x) contains supplementary material, which is available to authorized users.

## Background

A group of transcription factors constituting the retinal determination gene network (RDGN) is involved in the development of many types of animal eyes [1], and these factors probably already existed (albeit with different functions) at the bilaterian base [2]. One of the conserved and essential transcription factors in eye development is *pax6*, which is required for the formation of the retina in vertebrates [3]. In flies, the *pax6* ortholog *eyeless* (*ey*) and the pax gene *eye gone* are necessary to build the entire eye disc (reviewed by [4]). Additionally, *ey* and another *pax6* ortholog, *twin of eyeless* (*toy*), are involved in the development of ocelli but not of the Bolwig organ (the larval eye of some flies) (reviewed by [4]). *pax6* expression is also present in the early eye anlagen in cephalopods [5,6], planarians [7], nemerteans [8], polychaetes [9], the wasp *Nasonia vitripennis* [10], and the onychophoran *Euperipatoides kanangrensis* [11]. *pax6* is expressed in putative larval sensory cells in the calcisponges [12]. Nevertheless, it is not universally expressed during bilaterian visual system development. *pax6* expression is lacking in eyes or visual organs of an adult polychaete worm (*Platynereis dumerilii*) [9], a myriapod (*Glomeris marginata*) [13], amphioxus (*Branchiostoma floridae*) [14], and the horseshoe crab (*Limulus polyphemus*) [15]. The earliest target genes of *ey* have been categorized as ‘early retinal genes’ because their temporal expression extends from stages including the undifferentiated eye primordium to the differentiating retina [4]. This group of RDGN genes includes the *six1/2* homeobox gene *sine oculis* (*so*), *optix* orthologs or *six3* homeobox genes, the nuclear haloacid dehalogenase group phosphatase eyes absent (*eya*), and the Ski/Sno-related transcriptional co-factor dachshund (*dac*) [16]. The *eya* gene is critical for normal eye development in *Drosophila* [17]. *atonal* (*atn*), a proneuronal gene for photoreceptors in *Drosophila*, is a downstream RDGN element [18,19]. In the *Drosophila* eye disc, selection of R8 photoreceptors requires *atn* gene expression in a subset of proneural cells [19]. Additionally, formation of ocelli requires *atn* function [19]. Another crucial gene with an equally conserved role in eye and photoreceptor cell development is the *orthodenticle/otx* gene. In vertebrates, *otx* orthologs are required for the formation of many retinal cell types [20]. They are also expressed in the photoreceptors of the fly ommatidia, ocelli, and Bolwig organ [21]. The expression of the *otx* gene has also been documented in eyes and anterior neurogenic regions of different animals such as *Dugesia japonica* (planarian) [22], *Parhyale hawaiensis* (amphipod) [23], *Euscorpius flavicaudis* (scorpion), *Tegenaria saeva* (spider) [24], and *E. kanangrensis* (onychophoran) [11]. In *Tribolium castaneum*, two *otd*-related genes are present [25]. One of the two genes is expressed in a broad anterior stripe in the blastoderm, and the second gene is expressed in more limited subsets of cells in the anterior brain [25]. Other transcription factors involved in eye development belong to the *six* family of homeodomain proteins. In *Drosophila* [26], in planarians [27], and in the polychaete *P. dumerilii* [9], *six1/2* orthologs have an early role in eye specification. The vertebrate *six3* gene plays a pivotal role in vertebrate eye formation [28]. It is also involved in polychaete eye formation [9] but shows no expression in either the developing planarian [29] or the onychophoran eyes [11].

**Figure 1:**
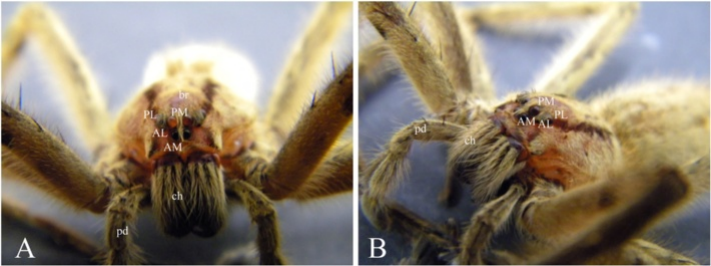
Image of a *C. salei* male showing the details of prosoma **(A)** frontal view and **(B)** lateral view. AL anterior-lateral eye, AM anterior-median eye, *br* brain, *ch* chelicerae, *pd* pedipalp, PL posterior-lateral eye, PM posterior-median eye.

The ctenid spider *C. salei* [30] *-* a large wandering spider from Central America - has been used as a model in evo-devo studies for many years [31], and recently, its complete development has been described in detail [32]. It has four pairs of well-developed eyes including one pair of principal eyes (PEs; or anterior-median eyes (AME)) and three pairs of secondary eyes (SEs) (posterior-median (PME), anterior-lateral (ALE), and posterior-lateral eyes (PLE)) (Figure 1A,B). The PEs of *C. salei* consist of a single layer of three to four rhabdomeric photoreceptors [33]. The major anatomical difference between PEs and SEs lies in the photoreceptor rhabdomeres, that is, everted (directed towards the lens) in the PE retina but inverted in the SE retina [33]. In addition, the retinas of PEs are mobile, using two special eye muscles, whereas the SE retinas are immobile [34]. Spiders exhibit three optic neuropils: all photoreceptors of each retina project towards their individual first optic neuropil (ON1); ON1 neurons send retinotopic projections to their individual secondary neuropil (ON2); the third optic neuropil (ON3) is common for all SEs, but a different area serves as the ON3 of the PEs [35-37]. The ON3 of PEs is part of a complex neuropil termed arcuate body [38] or central body [39]; the ON3 serving all SEs was [40] named mushroom body by Hanström [39]. ON1 and ON2 are equivalent to insects’ lamina and medulla, respectively [36].

Although the innervation and physiology of the eyes of adult *C. salei* have been extensively studied, the literature describing their detailed development is limited [32,34,38]. Doeffinger *et al*. [38] described the formation of the optic lobes during the embryonic development of *C. salei*. The onset of optic lobe formation is initiated as the bilateral grooves form in the lateral-most parts of the head neuroectoderm at 160 to 180 h after egg laying (hAEL). This structure is known as lateral furrows (*lf*) [32,38]. Later, the lateral grooves assume a kidney-like form at 220 hAEL [32,38]. At 250 hAEL, the lateral invagination has separated from the surface ectoderm, which most likely occurs by the partitioning of the grooves into a dorsal and a ventral vesicle [38]. Doeffinger *et al*. [38] assumed that these vesicles give rise to the optic ganglia of both lateral eyes (PLE and ALE). Later, the ventromedial neuroectoderm, adjacent to the lateral invaginations, invaginates and forms two vesicles. These, in turn, give rise to the optic ganglia of both median eyes (AME and PME [38]). Due to inconsistencies and incompleteness of the available literature on eye development in *C. salei*, we provide a more elaborate description of the ontogenesis of this spider’s optic neuropils and eye vesicles.

So far, the molecular mechanisms underlying eye development have not been studied in any spider species. Studying the molecular development of spider eyes is particularly important because they belong to a basal arthropod group, chelicerates, that are relatively understudied regarding molecular processes. Furthermore, spiders possess multiple eye types with different ontogeny and evolutionary history. Potential differences in the molecular patterning of spider eye types can teach us about the evolution of genetic networks in development. Except for a report on the lack of *pax6* and *atn* expression in the developing eyes of the xiphosuran horseshoe crab *Limulus* [15], the molecular mechanism of eye development in chelicerates remains unknown. We used next-generation RNA sequencing to amplify and characterise candidate genes for eye development in the spider *C. salei* and tested their expression pattern using whole-mount *in situ* hybridisation during embryonic development.

## Methods

### Animal husbandry and staging

Adult *C. salei* were kept in the breeding stocks in our laboratory animal facility in glass jars at 27°C and a relative humidity of 70% to 80%. They were fed once a week with flies. The cocoons were opened, and relevant embryonic stages (Table 1) were fixed for *in situ* hybridisation, antibody staining, or preserved in Trizol for RNA extraction.

**Table 1:** **Embryonic stages of** ***C. salei*** **according to Wolf and Hilbrant (2011) that are relevant for eye formation**

**Stage**	**Hours after fertilisation**	**Days**	**Name of stage**	**Description of stage relevant to eye formation**
Stage 11	100 to 130	4-5-6	Prosomal limb buds	Progression in the formation of bilateral cheliceral lobes
Stage 12	160 to 180	6-7-7.5	Lateral furrow	Formation of kidney-shaped folds lateral to stomodaeum (*lf*)
Stage 14	160 to 180	6-7-7.5	Inversion I	Migration of lateral subdivision (*ls*) in the direction of *lf* and partly covering it
				Formation of a crescent-shaped anterior furrow (*af*)
Stage 16	180 to 220	7.5-9	Inversion III	Tissue from *ls* completely covers the *lf*
				The growth of medial subdivisions (*ms*) anteriorly, partly covering the *af*
Stage 18	180 to 220	7.5-9	Prosomal shield	Growth of the rim of precheliceral lobes in the direction of mouth opening
				Formation of eye vesicles
Stage 20	220 to 280	9-11	Ventral closure	Distinct brain regions such as optic ganglia are evident, all four pairs of eyes are formed but not pigmented

### Transcriptome analysis and gene sequencing

Total RNA extraction was carried out using Trizol reagent according to manufacturer’s instructions from mixed embryonic stages (Life Technologies, Carlsbad, CA, USA). The extracted RNA was sent to Genecore (EMBL, Heidelberg, Germany) for sequencing (Illumina hi-seq, paired-end 100 bp, not normalised). Following quality filtering of reads and *de novo* transcript assembly using Velvet and Oases v0.2.08 (PMID: 22368243), we searched the resulting transcript database for matches to proteins downloaded from NCBI. BLAST searches and sequence analysis were done with the computer programme Geneious versions 5.6.6-7.1.5 created by Biomatters (http://www.geneious.com/). Primers were constructed based on sequences found in the assembled transcriptome database using the software primer3 [41]. The primers used are listed in Additional file 1. All genes were amplified and sequenced from embryonic cDNA for confirmation (Additional file 2).

### Orthology assignment

We used molecular phylogenetic methods to accurately assign the orthology of the genes. The accession numbers of the genes are provided in Tables in Additional files 3-7. Sequences were aligned using the programme ClustalW and MUSCLE implemented in the Geneious programme (http://www.geneious.com/). Bayesian inference on amino acid data using MrBayes v. 3.1.1 was applied for orthology analysis [42,43].

### Fixation of embryos and *in situ* hybridisation and microscopy

Fixation of embryos and *in situ* hybridisation was carried out according to Damen and Tautz [44]. After *in situ* hybridisation, the embryos were counter-stained either with SYBR green (Invitrogen, Waltham, MA, USA) or Hoechst (Sigma-Aldrich, St. Louis, MO, USA). The yolk was removed by micro-needles and hairs; the embryos were flat-mounted before taking photographs with an Olympus BX51 microscope (Olympus, Tokyo, Japan) using Cell^D software.

## Results

### Eye development

**Figure 2:**
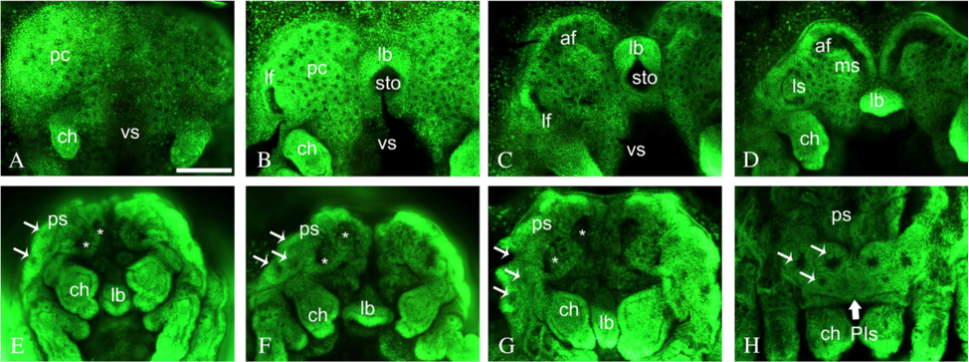
Nuclear staining of different embryonic stages explained in Table 1. **(A)** prosomal limb buds; **(B)** lateral furrow; **(C)** inversion I; **(D)** inversion III; **(E-G)** different stages of prosomal shield stage; **(H)** ventral closure. *ch* chelicerae, *lb* labrum, *lf* lateral furrow, *ls* lateral subdivision, *ms* median subdivision, *pc* precheliceral region or precheliceral lobes, *ps* prosomal shield, *sto* stomodaeum, *vs* ventral sulcus. Scale bar 200 μm.

In *C. salei*, the brain ontogeny starts with a series of depressions that fuse together and form deep invaginations. The first differentiation of the precheliceral region and the first event in optic system formation is the formation of a pair of lateral grooves, termed lateral furrows (*lf*), laterally on the stomodaeum at 160 to 180 hAEL (Table 1 stage 12 according to [32]; Figure 2A,B). *lf* leads to formation of a lateral subdivision (*ls*) (Table 1 stage 14 according to [32]; Figure 2C,D). Lateral furrow formation is accompanied by a median invagination. The latter is deeper and leads to a median subdivision (*ms*, Figure 2D). The *ls* migrates towards the *lf*, partly covering it in the middle, forming a figure-eight-like shape (Figure 2D). In the anterior region of the precheliceral lobes, an anterior furrow (*af*) or semi-lunar groove forms (Figure 2C,D). Later, at 180 to 220 hAEL, the *lf* is almost completely covered by the tissue from *ls*, presumably forming the anlagen of ONs of the lateral eyes ([38]; Figure 2D). The *ms* grows anteriorly, partly covering the *af* (Table 1 stage 16 according to [32]; Figure 2D). The rim of the precheliceral lobes grows medially towards the mouth opening and starts to cover the brain; this cover is termed the prosomal shield (Table 1 stage 18 according to [32]; Figure 2E,F,G). The eye vesicles of the SEs already form on the prosomal shield before it has reached the position of the optic neuropils, and the eye vesicles migrate with the prosomal shield, eventually ending up on top of the optic neuropil anlagen (Figure 2F,G). At about 180 to 200 hAEL, two pairs of vesicles form in the median subdivision (Table 1 stage 18 according to [32]; Figure 2E,F,G). These vesicles possibly form the optic neuropils (ON 1 to 2) of the posterior- and anterior-median eyes. As mentioned above, the eye vesicles of SEs have formed in the prosomal shield, migrating towards the mouth to cover the optic neuropils. In other spiders (*Heteropoda venatoria* and *Alopecosa spec*.), the AMEs or PEs do not form by vesicle invagination but by swelling of cells from the ectoderm in front of the prosoma [34]. The present investigation confirms that this is the case also in *C. salei* (Figure 2), where the AMEs or PEs form in the rims of the prosomal shield from a median position and move forward by the progress of the prosomal shield (Figure 2H). The dorsal ridge of the *af* then fuses with the posterior part of the brain, probably forming the arcuate or central body, which is the third neuropil of the PEs (see the ‘Background’ section). The remaining part of the *ms* probably participates in the formation of ON3 of SEs or the so-called mushroom bodies (Table 1 stage 20 according to [32]). We performed anti-acetylated tubulin antibody staining in order to follow the nerve fibres forming from ONs. This staining demonstrated that no optic fibres form until stage 18 or the prosomal shield stage (Additional file 2). After the ventral closure stage (Additional file 2) due to cuticle formation, whole-mount antibody staining was no longer technically possible.

### Orthology of the genes

#### Phylogenetic analyses of *Cs-pax6* genes

We found two orthologs of the *pax6* gene named *Cs-pax6a* and *Cs-pax6b*. Similar to Yang *et al*. [16], gene tree reconstruction of the paired domain, homeodomain, and the intervening conserved decapeptide sequence show monophyly of arthropods, lophotrochozoans, and chordates (Additional file 3). Additionally, *ey* and *toy* each form a monophylum (pink and yellow box in Additional file 3), except for *Anopheles gambiae ey*, which groups with *toy*. The Bayesian likelihood tree categorises *Cs-pax6a* and *Cs-pax6b* to the *ey* and *toy* monophyla, respectively (Additional file 3). The sequence of *Cs-pax6a* and the theridiid (cobweb) spider’s *pax6.1* from *Parasteatoda tepidariorum* (*Pte-pax6.1*) (Schomburg *et al*. submitted) form a sister group, and *Cs-pax6b* together with *Pte-pax6.2* and *Limulus pax6* (*Lpo-pax6*) form a chelicerate monophylum (Additional file 3). The tree shows that duplication of pax6 possibly occurred before the divergence of chelicerates.

#### Phylogenetic analyses of *Cs-dac* genes

Two orthologs of the *dachshund* gene were found in the spider. The phylogenetic tree of *dachshund* genes, with *ski* genes as an outgroup, shows one monophylum each of vertebrates (pink box in Additional file 4) and protostomes (yellow box in Additional file 4). Within the arthropods, arachnids’ *dac* orthologs appear as a monophylum, showing that spiders’ *dac* duplication occurred more recently, probably at the base of arachnids (Additional file 4). *Cs-daca* forms a sister group to *Pte-dac1*, and *Cs-dacb* forms a sister group to *Pte-dac2* together with both of the eresid (velvet) spider *Stegodyphus mimosarum*’s *Smi-dac* orthologs.

#### Phylogenetic analyses of *Cs-atn* genes

Two orthologs of the *atonal* gene were found in the spider. We made a phylogenetic reconstruction of the *atonal* genes together with other basic helix-loop-helix family genes to clearly demonstrate whether our *atonal* gene group with *atonal* genes from the other species. The tree shows a monophylum for *neurogenin* (*ngn*), *Target of pox neuron* (*TAP*), *Scleraxis* (*scx*), and *atonal* and *amos* genes (yellow box in Additional file 5). The few exceptions are discussed in Additional file 5. The chelicerates’ *atonal* genes form a monophylum. *Cs-atha* and *Pte-ath1* as well as *Cs-athb* and *Pte-ath2* each form sister groups (Additional file 5). *Limulus* has only one *atonal* ortholog (*Lpo-ath*) that forms a sister group to both *Cs-atha* and *Pte-ath1* (Additional file 5). This suggests that *atonal* duplication occurred at the base of arachnids.

#### Phylogenetic analyses of *Cs-otx* genes

We found two orthologs of the *otx* gene in the spider. We constructed the *otx* tree using *aristaless (arx)* as an outgroup (Additional file 6). The two orthologs of *otx* in chelicerates (named *otxa* and *otxb* in *Cupiennius*) do not correspond to *otx1* and *otx2* of vertebrates, showing that the duplication of the genes in chelicerates occurred independently from duplication of *otx* in vertebrates. Arthropods’ *otx* genes do not form a monophylum, and the position of the two chelicerate orthologs within the arthropods is unresolved (Additional file 6).

#### Phylogenetic analyses of *Cs-six* genes

The phylogenetic reconstruction of the *six* family produced a monophylum for each of the *six1/2* (Additional file 7 yellow box), *six3* (Additional file 7 pink box), and *six4* genes (Additional file 7 blue box). The exception is the *Apis mellifera six2* gene (which is grouped with *six4* genes and is possibly incorrectly assigned to *six2*). We found two orthologs of *six1* and two orthologs of *six 3* in the spider. The two orthologs of spiders’ *six1* form a sister group to *six1* of other arthropods. *Cs-six1a* forms a sister group to the two other spider *six1* genes, namely *Pte-so1* and *Smi-six1a. Cs-six1b* forms a sister group to the spider *Smi-six1b*, and these two both form a sister group to the spider *Pte-so2*. This shows that the duplication of six genes probably occurred at the base of the arachnid group. The tree is less well resolved in the case of *six3* genes. Arachnid’s *six3* genes appear as a monophylum, but the two orthologs do not show a sister relationship. The two copies of *Cs-six1* show high similarity of the paired domain, homeodomain, and decapeptide sequences, but areas outside of these domains are unconserved. This results in an only 49% sequence similarity globally; the same holds true for *Cs-six3*, with 60% similarity globally.

### Gene expression patterns

Table 2 summarizes the expression pattern of each gene in different stages of development. Expression has been mainly tested in prosomal limb bud, lateral furrow, prosomal shield, and ventral closure stages. Some of the genes are expressed in structures that are not directly related to the eyes. Examples are expression of *Cs-pax6a* in the ventral neuroectoderm (not shown) or expression of the genes in other invaginating neural precursor (INP) groups or labrum, which are not discussed here.

**Table 2:** **Expression of** ***C. salei pax6*** **,** ***eya*** **,** ***atn*** **,** ***otx*** **,** ***six1*** **, and** ***six3*** **in different embryonic stages**

**Name of gene**	**Embryonic stages**			
	**Prosomal limb buds**	**Lateral furrow**	**Prosomal shield**	**Ventral closure**
*Cs-pax6a*	Two symmetric patches at the middle of precheliceral lobes	Medial side of *lf*	*ms* in the area of future mushroom body and medial side of *lf* and other INPs	Vesicles of both PEs
*Cs-pax6b*	_	An oblique figure-eight-shaped patch in central part of precheliceral lobes and median side of *lf*	*ms* in the area of future mushroom body, median INPs	median INPs
*Cs-eya*	Two patches anterior and posterior of future forming *lf* in precheliceral lobes	Anterior and posterior of *lf*	Future area of arcuate body of *af* and forming secondary eye vesicles in the rim of prosomal shields	Vesicles of SEs
*Cs-daca*	_	_	In the rim of prosomal shields (forming vesicles of lateral eyes)	_
*Cs-dacb*	Lateral and medial of *lf* and INPs in the place of forming *af*	Prosomal shield and medial INPs	In the rim of prosomal shields around the forming eye vesicles	In the prosomal shields around the forming eye vesicles
*Cs-atna*	In the lateral-most sides of precheliceral lobes, at the site of forming *lf*	In the forming *ms* and two symmetric patches under forming *af*	In the INPs of precheliceral lobes and future mushroom body area of *af*	_
*Cs-atnb*	_	Median side of *lf* and few INPs in the area of *ms*	Median side of *lf* and future mushroom body area of *af*	INPs of precheliceral lobes
*Cs-otxa*	Two symmetric patches in the lateral and median sides of precheliceral lobes	*ls* and INPs of *ms*	Median INPs of precheliceral lobes	Vesicle of PLEs
*Cs-otxb*	Two symmetric patches in the centre of precheliceral lobes	*lf*, INPs of *ms* and other INPs	Future mushroom body area of *af* and INPs at median and lateral sides of precheliceral lobes	Vesicles of all SEs
*Cs-six1a*	_	Lateral side of *lf*	In the rim of prosomal shield at the area of forming SEs	All SE vesicles
*Cs-six1b*	_	Median region of *lf* and INPs of *ms*	Future area of arcuate body of *af* and in the rim of prosomal shields (forming vesicles of posterior eyes) and future mushroom body area of *af*	Vesicles of PMEs and PLEs, faint expression in the vesicles of PEs
*Cs-six3a*	3 pairs of patches (two lateral and one medial) in the precheliceral lobes	Median side of *lf* and future mushroom body area of *af*	In the rim of prosomal shield (forming eye vesicles of lateral eyes) and ON of PE	Vesicles of PLEs and PMEs and ON of PE
*Cs-six3b*	_	In the *ls* and *ms* in the area of future mushroom body of *af*	Forming vesicles of SEs in the rim of prosomal shield and median part of *af* in the region of future mushroom body of *af*	All SE vesicles

#### Expression of *Cs-pax6a*

**Figure 3:**
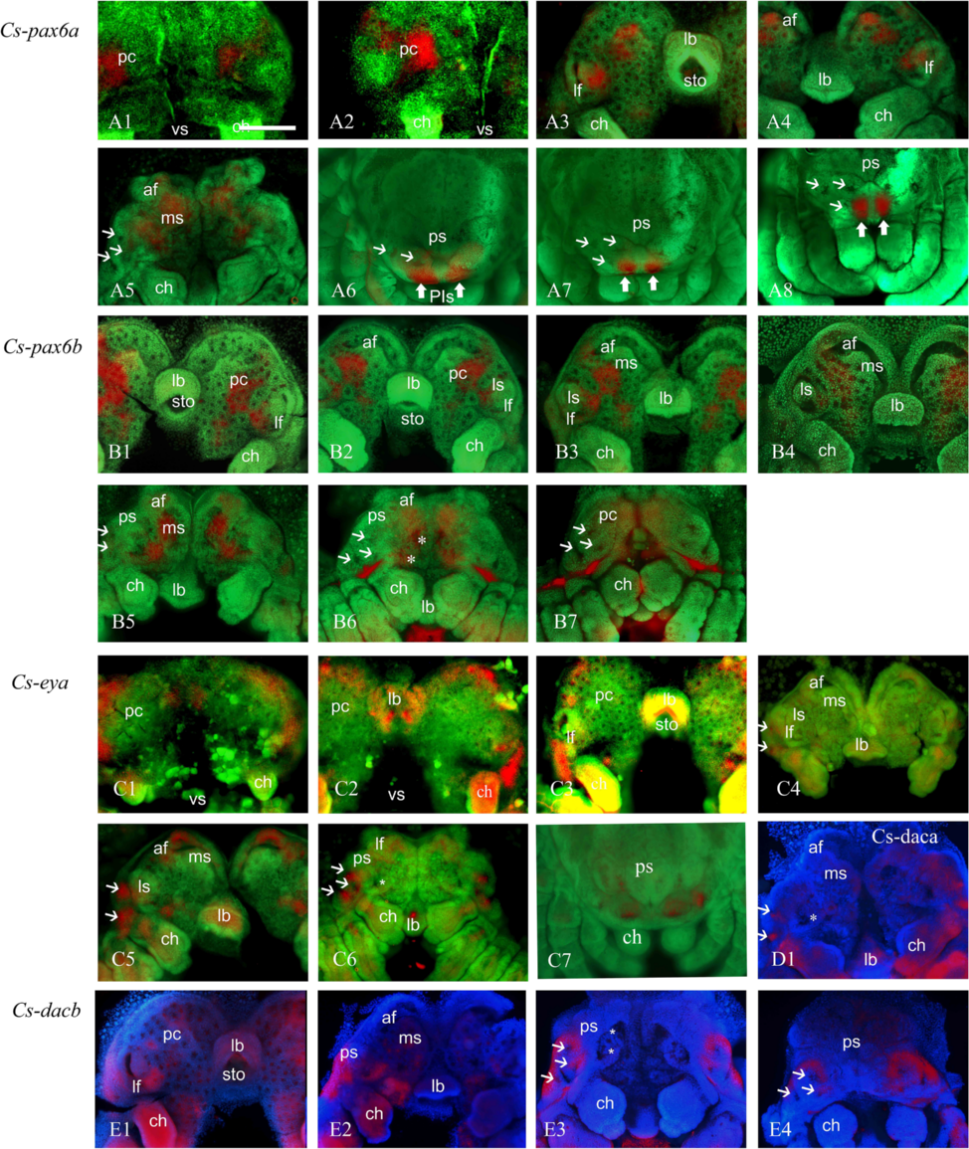
Expression of *C. salei pax, eya,* and *dac* in different stages of development. Embryos are flat-mounted and counter stained with SYBR green or Hoechst. **(A1-A8)** *Cs-pax6a*, **(B1-B7)** *Cs-pax6b*, **(C1-C6)** *Cs-eya*, **(D1)** *Cs-dac1*, **(E1-E4)** *Cs-dac2.* (A1), (A2), (C1), (C2) prosomal limb buds; (A3), (B1), (C3), (E1) lateral furrow; (A4), (B2), (C4) inversion I; (B3), (B4) inversion III; (A5), (B5), (B6), (C5), (C6), (D1), (E2), (E3) prosomal shield; (A6), (A7), (A8), (B7), (C7), (E4) ventral closure. Thin arrows: secondary eye vesicles, Thick arrows: principal eye anlagen, asterisk: ON of median eyes. *ch* chelicerae, *lb* labrum, *lf* lateral furrow, *ls* lateral subdivision, *ms* median subdivision, *pc* precheliceral region or precheliceral lobes, *ps* prosomal shield, *sto* stomodaeum, *vs* ventral sulcus. Scale bar 200 μm.

In the prosomal limb bud stage, *Cs-pax6a* is expressed in two symmetric patches at the middle of the precheliceral lobes (Figure 3A1,A2). In the lateral furrow stage, expression is detected in medial side of *lf* (Figure 3A3). This expression pattern is consistent in the inversion I stage (Figure 3A4). The gene is expressed in *ms* in the area of future mushroom body and on the medial side of *lf* and other INPs in the prosomal shield stage (Figure 3A5). In the ventral closure stage, the gene is expressed in vesicles of both PEs (Figure 3A6,A7,A8).

#### Expression of *Cs-pax6b*

*Cs-Pax6b* is not expressed in the prosomal limb bud stage. The first sign of expression pattern is evident in the lateral furrow stage, where an oblique figure-eight-shaped patch is detectable in central part of the precheliceral lobes and median side of *lf* (Figure 3B1). This expression remains the same in the inversion I stage (Figure 3B2) and inversion III stage (Figure 3B3,B4). In the prosomal shield stage, the gene is expressed in *ms* in the area of the future mushroom body and median INPs (Figure 3B4,B5,B6). This expression pattern includes expression of the gene in the anlage of ONs of median eyes (asterisk in Figure 3B6). In the ventral closure stage, the gene is expressed in median INPs.

#### Expression of *Cs-eya*

In the prosomal limb bud stage, *Cs-eya* is expressed in two patches anterior and posterior of future *lf* in precheliceral lobes (Figure 3C1,C2). The expression is detected anterior and posterior of *lf* in the lateral furrow stage (Figure 3C3). This expression is consistent in the inversion I stage (Figure 3C4). The future area of the arcuate body of *af* and the forming secondary eye vesicles in the rim of prosomal shields are the areas which express *Cs-eya* in the prosomal shield stage (Figure 3C5,C6). In the ventral closure stage, the gene expression is seen in the vesicles of SEs (Figure 3C7).

#### Expression of *Cs-daca*

*Cs-daca* is expressed only in the rim of prosomal shields (forming vesicles of lateral eyes) in the prosomal shield stage (Figure 3D1).

#### Expression of *Cs-dacb*

In the lateral furrow stage, *Cs-dacb* is expressed lateral and medial of *lf* and INPs at the site of forming *af* (Figure 3E1). In the prosomal shield stage, the gene is expressed in prosomal shield and medial INPs (Figure 3E2,E3). The gene expression is observed in the rim of prosomal shields around the forming eye vesicles in the ventral closure stage (Figure 3E4).

#### Expression of *Cs-atna*

**Figure 4:**
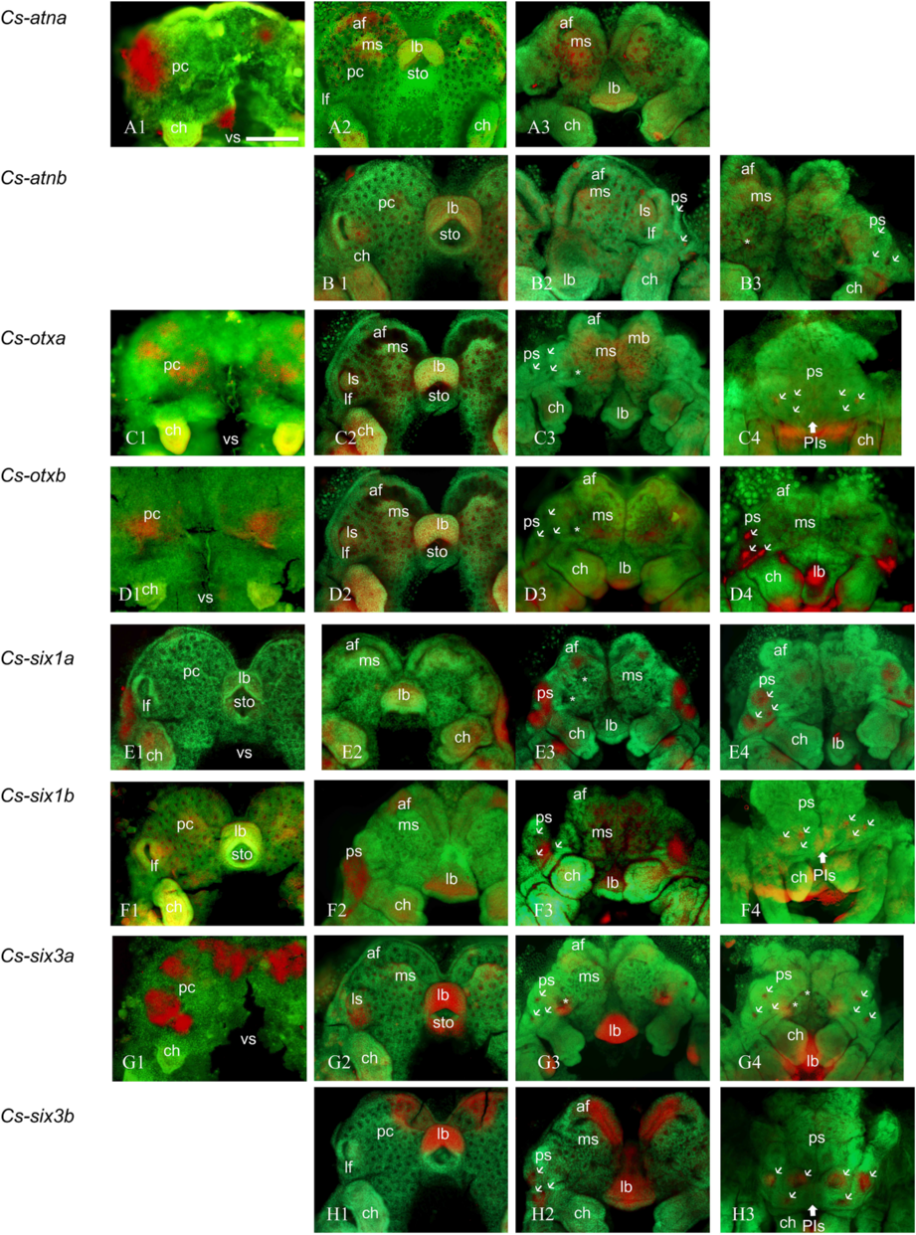
Expression of *C. salei atn, otx, six1,* and *six2* in different stages of development. Embryos are flat-mounted and counter stained with SYBR green. **(A1-A3)** *Cs-atna*, **(B1-B3)** *Cs-atnb*, **(C1-C4)** *Cs-otxa*, **(D1-D4)** *Cs-otxb*, **(E1-E4)** *Cs-six1a*, **(F1-F4)** *Cs-six1b*, **(G1-G4)** *Cs-six3a*, **(H1-H3)** *Cs-six3b*. (A1), (C1), (D1), (G1) prosomal limb buds; (A2), (B1), (E1), (F1), (G2), (H1) lateral furrow; (C2), (D2), (E2) inversion I; (F3) inversion III; (A3), (B2), (B3), (C3), (D3), (E3), (F3), (G3), (G4), (H2) prosomal shield; (C4), (D4), (E4), (F4), (H3) ventral closure. Thin arrows: secondary eye vesicles, Thick arrows: principal eye anlagen, asterisk: ON of median eyes. *ch* chelicerae, *lb* labrum, *lf* lateral furrow, *ls* lateral subdivision, *ms* median subdivision, *pc* precheliceral region or precheliceral lobes, *ps* prosomal shield, *sto* stomodaeum, *vs* ventral sulcus. Scale bar 200 μm.

In the prosomal limb bud stage, *Cs-atna* is expressed in the lateral-most sides of precheliceral lobes, at the site of forming *lf* (Figure 4A1). In the lateral furrow stage, the gene expression is detected in the forming *ms* and two symmetric patches under forming *af* (Figure 4A2). The gene is expressed in the INPs of precheliceral lobes and future mushroom body area of *af* in the prosomal shield stage (Figure 4A3). The gene is not expressed in the ventral closure stage.

#### Expression of *Cs-atnb*

*Cs-atnb* is not expressed in the prosomal shield stage. The first sign of expression appears at the lateral furrow stage in median side of *lf* and few INPs in the area of *ms* (Figure 4B1). In the prosomal shield stage, the gene is expressed in median side of *lf* and future mushroom body area of *af* (Figure 4B2,B3). The gene is not expressed in the ventral closure stage (data not shown).

#### Expression of *Cs-otxa*

In the prosomal limb bud stage, *Cs-otxa* is expressed in two symmetric patches in the lateral and median sides of precheliceral lobes (Figure 4C1). In the lateral furrow stage, the gene is expressed in *ls* and INPs of *ms* (Figure 4C2). The expression is detected in median INPs of precheliceral lobes in the prosomal shield stage (Figure 4C3). In the ventral closure stage, the pattern is detected in the vesicle of PLEs.

#### Expression of *Cs-otxb*

In the prosomal limb bud stage, *Cs-otxb* is expressed in two symmetric patches in the centre of precheliceral lobes (Figure 4D1). In the lateral furrow stage, the pattern is detected in *lf*, INPs of *ms*, and other INPs (Figure 4D2). In the prosomal shield stage, the gene is expressed in the future mushroom body area of *af* and INPs at median and lateral sides of precheliceral lobes (Figure 4D3). The pattern is detectable in the vesicles of all SEs in the ventral closure stage (Figure 4D4).

#### Expression of *Cs-six1a*

*Cs-six1a* is not expressed in the prosomal limb bud stage (data not shown). In the lateral furrow stage, the first sign of expression pattern is detectable in lateral side of *lf* (Figure 4E1). The expression is the same in the inversion I stage (Figure 4E2). In the prosomal shield stage, the gene is expressed in the rim of the prosomal shield at the area of forming SEs (Figure 4E3). In the ventral closure stage, the gene is expressed in the vesicles of all SEs (Figure 4E4).

#### Expression of *Cs-six1b*

*Cs-six1b* is not expressed in the prosomal shield stage (data not shown). The median region of *lf* and INPs of *ms* is the areas that express *Cs-six1b* in the lateral furrow stage (Figure 4F1). The gene is expressed in lateral side of *lf* in the inversion III stage (Figure 4F2). The gene expression is detected in future area of the arcuate body of *af* and in the rim of prosomal shields (forming vesicles of posterior eyes) and future mushroom body area of *af* in the prosomal shield stage (Figure 4F3). In the ventral closure stage, vesicles of PMEs and PLEs show expression of *Cs-six1b*. In addition, we detected a faint expression of the gene in the PE vesicles (Figure 4F4).

#### Expression of *Cs-six3a*

In the prosomal limb bud stage, three pairs of patches (two lateral and one medial) in the precheliceral lobes express *Cs-six3a* (Figure 4G1). In the lateral furrow stage, the gene is expressed in median side of *lf* and future mushroom body area of *af* (Figure 4G2). The rim of the prosomal shield (forming eye vesicles of lateral eyes) and ON of PE are the places that express *Cs-six3a* in the prosomal shield stage (Figure 4G3). In the ventral closure stage, the gene expression appears in the vesicles of PLEs and PMEs and ON of PE (Figure 4G4).

#### Expression of *Cs-six3b*

*Cs-six3b* is not expressed in the prosomal limb bud stage. The expression in the lateral furrow stage occurs in the *ls* and *ms* in the area of the future mushroom body of *af* (Figure 4H1). In the prosomal shield stage, the gene is expressed in forming vesicles of SEs in the rim of the prosomal shield and in the region of the future mushroom body of *af* (Figure 4H2). In the ventral closure stage, the gene is detectable in all SE vesicles (Figure 4H3).

#### Expression in eye vesicles

**Figure 5:**
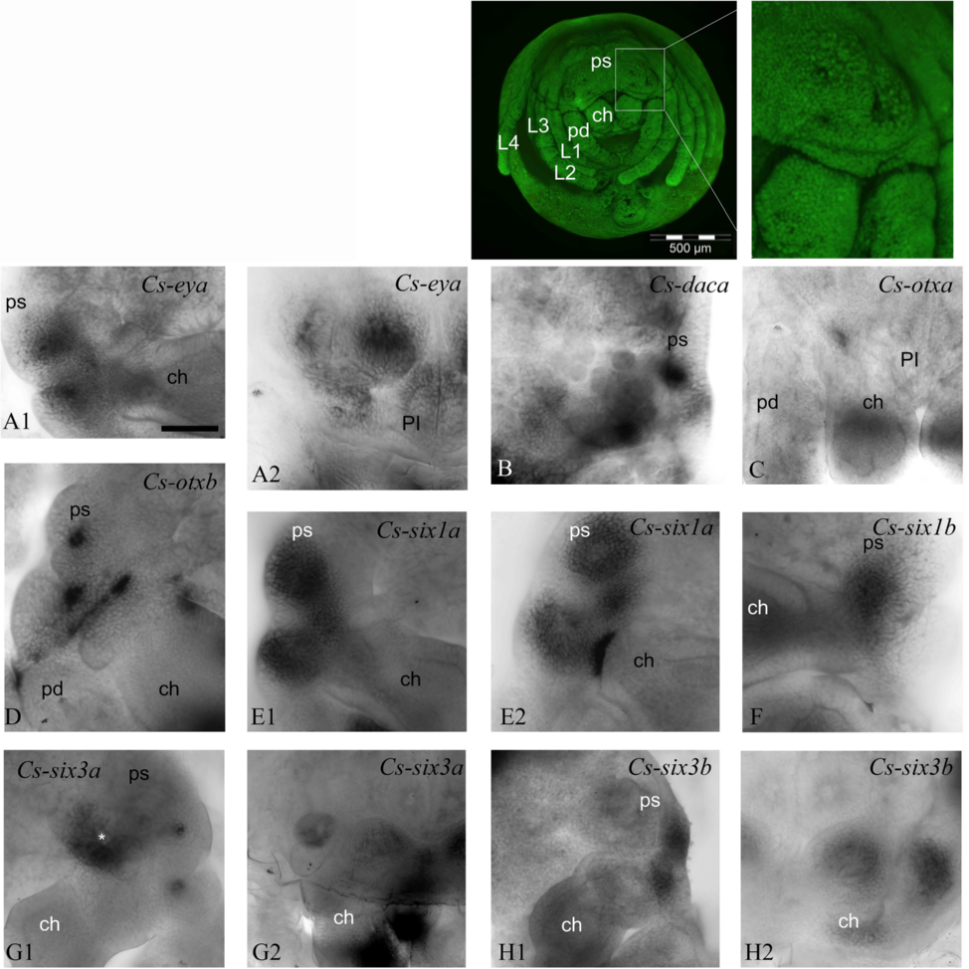
Expression of the RDGN elements in eye vesicles in prosomal shield and ventral closure stages. **(A1), (A2)** *Cs-eya*; **(B)** *Cs-daca*; **(C)** *Cs-otxa*; **(D)** *Cs-otxb*; **(E1), (E2)** *Cs-six1a*; **(F)** *Cs-six1b*; **(G1), (G2)** *Cs-six3a*; **(H1), (H2)** *Cs-six3b*. (A1), (B), (E1), (F), (G1), (H1), prosomal shield; (A2), (C), (G2), (H2), ventral closure. *ch* chelicerae*, pd* pedipalps, *ps* prosomal shield, asterisk: ON of median eyes. Scale bar 100 μm.

Several of the genes we studied are expressed in the forming vesicles of the eyes, which start at the prosomal shield stage (180 to 220 hAEL) and continue to the ventral closure stage (220 to 280 hAEL). The genes are expressed differentially in different vesicles (Figures 3 and 4). *Cs-pax6a* is expressed in the area where the anlagen of PEs delaminates (Figure 3A6,A7,A8). This is not the case for *Cs-pax6b*, which is expressed in two patches of INPs in *ms* (Figure 3B5), which later corresponds to the ON of the median eyes (Figure 3B6). In addition, *Cs-six1b* and *Cs-eya* show a faint expression pattern in the vesicles of PEs (Figures 4F4 and 5A2). *Cs-dacb* is not expressed in the eye vesicles themselves but in an area surrounding the vesicles of SEs (Figure 3E2,E3,E4). *Cs-eya* (Figure 3C5,C6; Figure 5A1,A2), *Cs-otxb* (Figure 4D4; Figure 5D), *Cs-six1a* (Figure 4E3,E4; Figure 5E1,E2), and *Cs-six3b* (Figure 4H2,H3; Figure 5H1,H2) are expressed in the forming SE vesicles, *Cs-daca* (Figure 3D1; Figure 5B) PLEs and ALEs, *Cs-otxa* in the vesicles of PLEs (Figure 4C4; Figure 5C), and *Cs-six3a* (Figure 4G3,G4; Figure 5G1,G2) and *Cs-six1b* in the vesicles of PMEs and PLEs (Figure 4F3,F4; Figure 5F).

## Discussion

### Expression around the lateral furrow

The expression pattern of most of the genes starts before the onset of eye development at the prosomal limb bud stage, 100 to 130 hAEL (Table 2, Figures 3 and 4). Some of the genes, however, do not show any expression at this stage (Table 2, Figures 3 and 4). At the prosomal limb bud stage, *Cs-pax6a* shows two symmetric patches of expression at the centre of the precheliceral area (Figure 3A1,A2); *Cs-eya* shows two symmetric patches of expression anterior and posterior of future forming *lf* (Figure 3C1,C2); *Cs-atha* shows two symmetric patches in the lateral-most part of precheliceral lobes at the site of future *lf* (Figure 4A1); *Cs-dacb* (Figure 3E1) and *Cs-otxa* (Figure 4C1) show two pairs of symmetric patches on the lateral and medial sides of the precheliceral lobes; *Cs-otxb* shows two symmetric patches in the middle of the precheliceral lobes (Figure 4D1); and *Cs-six3a* shows three pairs of expression patches (two in the lateral and one in the medial regions of the precheliceral lobes; Figure 4G1). These genes are later (160 to 180 hAEL) expressed around the area of *lf. Cs-pax6a* (Figure 3A3) and *b* (Figure 3B1,B2,B3,B4), *Cs-atnb* (Figure 4B1), *Cs-otxa* (Figure 4C2), *Cs-six1b* (Figure 4F1), and *Cs-six3a* (Figure 4G2) show overlapping expression in the median side of the lateral furrow where the lateral subdivision is forming. *Cs-eya* is visible as two symmetric pairs of expression areas anterior and posterior of *lf* (Figure 3C2,C3). *Cs-six1a* is expressed in the lateral side of *lf* (Figure 4E1). *Cs-six3b* shows no pattern of expression around *lf* at this stage (Figure 4H1).

### Expression in optic neuropils of the median eyes

*Cs-dacb* (Figure 3E1) and *Cs-six3a* (Figure 4G3) are the only two genes whose expression corresponds to the area of the forming first and second optic neuropil anlagen of the PEs. *Cs-pax6a* (Figure 3A5) and *Cs-pax6b* expression corresponds to the anlage of ONs of both median eyes (asterisks on Figure 3B6).

### Expression in the area of the mushroom body anlage of the anterior furrow

At 180 to 220 hAEL, the *ms* grows towards the *af* and partly covers it to form the mushroom body or ON3 of SEs. Several of the genes including *Cs-pax6a* (Figure 3A4,A5) and *b* (Figure 3B5), *Cs-atna* (Figure 4A3) and *b* (Figure 4B2), *Cs-otxa* (Figure 4C3) and *b* (Figure 4D3), *Cs-six1a* (Figure 4E3), and *Cs-six3a* (Figure 4G3) are expressed in this area at this stage.

### Expression in the area of the arcuate body anlage of the anterior furrow

*Cs-eya* (Figure 3C4,C5) as well as *Cs-six1b* (Figure 4F2) and *3b* (Figure 4H2) are expressed in the area of the anterior furrow in the region of the forming arcuate body or ON3 of PEs at 180 to 220 hAEL.

### Eye development and differential expression of the genes

**Figure 6:**
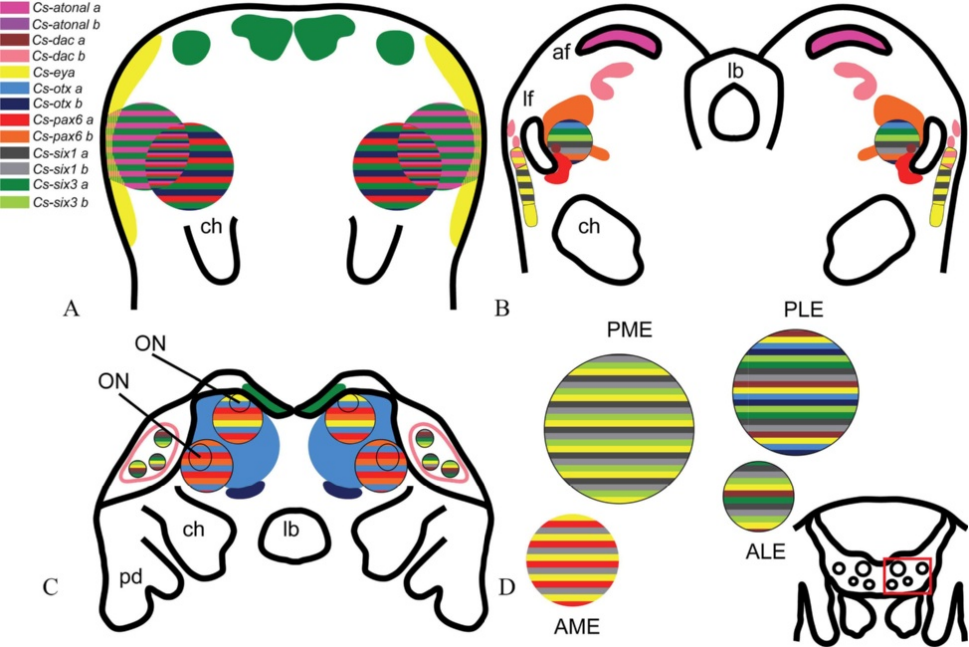
Scheme summarizing the expression of RDGN genes in the main embryonic stages and the vesicles of the eyes. **(A)** Prosomal limb buds. **(B)** Lateral furrow. **(C)** Prosomal shield. **(D)** Ventral closure. *af* anterior furrow, ALE anterior-lateral eyes, AME anterior-median eyes, *ch* chelicerae*, lb* labrum, *lf* lateral furrow, ON optic neuropil, *pd* pedipalps, PLE posterior-lateral eyes, PME posterior-median eyes.

Eye development in the spider *C. salei* is somewhat unconventional, initiating with the formation of the ON anlagen in the differentiating neuroectoderm. Anti-acetylated tubulin staining does not show any optic fibres forming until stage 18. This contradicts the description of ON formation by Doeffinger *et al*. [38], who note ONs already at 160 to 180 hAEL. We corroborate Doeffinger *et al*. in stating that the ‘anlagen’ of optic neuropils form from *lf*, medial invaginations, and *af*. ONs of the lateral eyes form earlier than the ONs of the median eyes. The ONs of the lateral eyes form with the *lf*, and the ONs of the median eyes form when two pairs of vesicles are separated from the median subdivision. Afterwards, *af* forms what will give rise to the arcuate body or ON3 of PEs; the growth of *ms* over the posterior part of *af* forms the mushroom body or ON3 of SEs. Only then do the vesicles of SEs form in the prosomal shield and start migrating over ON to make contact with them. This contradicts the observation of Doeffinger *et al*. [38], who assume that *lf* separates from the surface ectoderm and forms the optic vesicles of lateral eyes. This unconventional process of eye formation may explain why several of the RDGN genes initiate to show their expression in ON anlagen. At the prosomal limb bud stage, the area of the future lateral furrow is discernible by overlapping expression of several RDGN genes (Figure 6A). In addition to this area, *Cs-six3a* marks the area of the future anterior furrow (Figure 6A). *Cs-eya* and *Cs-six1a* mark the lateral-most side of the prosomal lobes, where the prosomal shield later forms (Figure 6A,B). In the ON anlagen of lateral eyes (lateral furrow), most of the elements of RDGN demonstrate expression around this region (Figure 6B). Expressions of several of the genes map to the area where lateral subdivision takes place (Figure 6B). *Cs-pax6a* and *Cs-pax6b* show complementary non-overlapping expression in this area (Figure 6B). In contrast, *Cs-pax6a* and *Cs-pax6b* have overlapping expression in the ONs of the median eye (Figure 6C). Different sets of genes are expressed in ON3 of PEs and SEs (Figure 6). In earlier stages of development, the expressions of RDGN genes in *P. tepidariorum* (with the exception of *Pte-so* genes) are similar to the expression of the *Cupiennius* homologs (Schomburg *et al*. submitted). In *Limulus*, *Lp-pax6* and *Lp-atn* are not expressed during the formation of different eyes [15]. According to our phylogenetic analyses, *Lp-pax6* cluster together with *Cs-pax6b* in the *toy* monophylum. *Cs-pax6b*, similar to *Lp-pax6*, is not directly expressed in the eyes, but rather in the ON of the median eyes and other brain regions. The same applies for *Cs-atn*, which has an expression similar to *Lp-atn* expression patterns in brain centres rather than directly in the eyes [15]. The lack of an *ey* ortholog in *Limulus* could reflect deficiency in PCR screening; this requires further investigation to finalize the possible role of *ey* in median eye development in *Limulus*.

**Figure 7:**
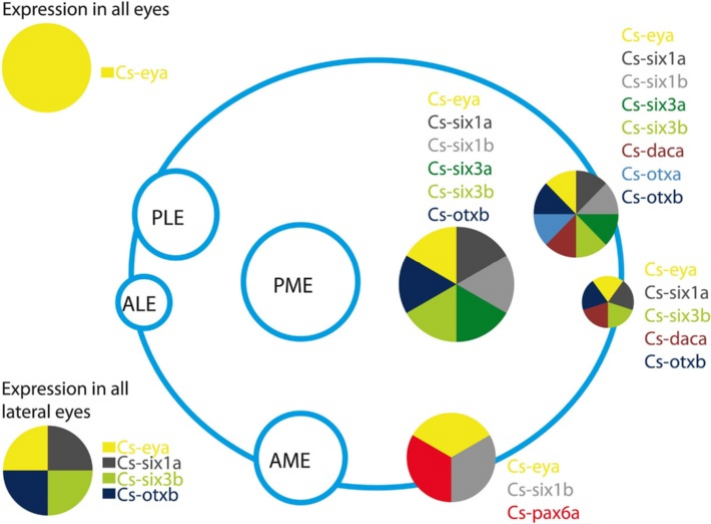
Schematic expression of the genes in the eye vesicles. ALEs anterior-lateral eyes, AMEs anterior-median eyes, PLEs posterior-lateral eyes, PMEs posterior-median eyes.

The RDGN genes also show differential expression in the eye vesicles, so that each developing eye can be distinguished by a specific set of transcription factors (Figures 7 and 6D). For example, *Cs-pax6a*, *Cs-six1b*, and *Cs-eya* determine the PEs (Figures 7 and 6D). This is the same as principal eye determination in the spider *P. tepidariorum*, with the difference that, in that species, *Pte-Otd* genes also are expressed in the PEs, which is not the case in *C. salei* (Schomburg *et al*. submitted). *Cs-eya*, *Cs-otxb*, *Cs-six1a*, *Cs-six3b,* and *Cs-daca* determine ALEs (Figures 7 and 6D). Excluding *otxb*, the same genes determine the ALEs in *P. tepidariorum*, with the difference that, in this spider, *Pt-so2* is also expressed in ALEs (Schomburg *et al*. submitted). The same set of genes as ALEs together with *Cs-otxa*, *Cs-six1b*, and *Cs-six3a* distinguish PLEs (Figures 7 and 6D). This is different from PLEs in *P. tepidariorum.* The only similar genes in this pair of eyes are *eya*, *six1a* or *so1*, and *six3a* or *six3.2. Cs-eya*, *Cs-otxb*, *Cs-six1a*, and *Cs-six1b* together with *Cs-six3a* and *Cs-six3b* determine PMEs (Figure 6 and 6D). In *P. tepidariorum*, in addition to these genes also *dac* orthologs are expressed in the PMEs. Each eye pair is apparently specified by a different combination of RDGN transcription factors, and recruitment of the genes in different eyes was at least partly independent in the two spiders. The reason for differential gene expression among the secondary eyes is probably the diverging function of the adult eyes combined with genetic drift [45]. The analysis of gene expression pattern, however, also makes it possible to distinguish the secondary from the principal eyes. The lateral eyes share the expression of four out of a total of eight genes expressed in secondary eyes, whereas all eyes (principal + secondary) have only one gene in common (Figure 7). To further assess how these genes interact with each other and how the gene networks have evolved in each eye, we aim to perform knockdown experiments of the key genes in each eye vesicle and follow how the expression of the other RDGN genes is affected.

### Origin of *ey* and *toy* in arthropods

Yang *et al*. (2009) suggested that the duplication generating *ey* and *toy* occurred before the diversification of the major arthropod subgroups Pancrustacea, Myriapoda, and Chelicerata because there are two *pax6* genes in the insects *Drosophila* and *Tribolium* and in the myriapod *Glomeris*. Our data support this hypothesis by demonstrating the existence of both homologs of *ey* (*Cs-pax6a* and *Pte-pax6.1*) and *toy* (*Cs-pax6b*and *Pte-pax6.2*) in the two arachnids, with possible different functions due to different gene expression patterns. The lack of two *pax6* genes in *Limulus* [15] may merely reflect technical deficiency of PCR screening.

### *pax*-dependent and *pax*-independent gene networks regulating principal and secondary eye formation

*ey* and *toy* are among the earliest selector genes expressed in the eye-antennal imaginal disc in *Drosophila*. Their expression becomes specific to the eyefield during the second larval instar (reviewed by [4]). Both are also present in the region of the eye disc, where the ocelli develop (reviewed by [4]). It remains unclear, however, whether this expression is down regulated once ocellus differentiation initiates. Expression of *toy* has not been observed in the Bolwig organ, and reports considering the expression of *ey* are conflicting (reviewed by [4]). In *Tribolium*, knockdown of *ey* and *toy* affects larval eye development strongly but adult eye development only mildly. Compound eye loss was reported in the combination of *ey*, *toy*, and *dac* knockdown [16]. The homolog of *ey* in the spider *Cs-pax6a* is expressed in the area of the lateral furrow and median subdivision, where most of the *ey* downstream genes in the RDGN are expressed. *Cs-pax6a* is the sole gene of those investigated that is expressed in the PE vesicles of *Cupiennius* (Figure 5)*.* Cs-eya and Cs-six1b show a weak expression in this pair of eyes (Figure 7). The need for *pax6* activation during PE development is consistent with the data that consider a universal role of *pax6* in animal eye development [46]. This expression in PEs may activate other genes such as *eya* and *six*. Future knockdown experiments may identify the molecules functioning downstream of *pax6* genes in the PEs. It is also possible that, in *Cupiennius*, RDGN genes downstream of *pax6* function only in the optic neuropil instead of the retina. Another possibility is that *Cs-pax6a* is involved in transcriptional control of eye differentiation in other ways, such as activation of opsin and the pigmentation process, as was also suggested in *Platynereis* eye development [9]. The direct role of the *pax6* gene in photoreceptor cell differentiation [46] clearly does not apply for all spider eyes. The SEs differentiate in the absence of *Cs-pax*. How then can the redundancy of *pax6* genes for SEs be explained? Co-existence of distinct eye types, differentiating with or without *pax6*, has also been shown for other groups, for example, adult eye differentiation in the absence of *pax6* in polychaetes [9], squid [6], horseshoe crabs [15], myriapods [13], and the Joseph cells and Hesse organs of lancet fish [14]. The difference between eye determination pathways in the PEs and SEs can also be explained by the ways in which the eyes develop. PE development involves swelling of the epidermis rather than vesicle formation and yields the so-called inverted eyes, which exhibit everse retina despite the reversal of the epidermal layer [47]. These two eye types are also functionally different: the forward-facing pair of PEs have narrow fields of view but high resolution that probably detect shape, whereas the three pairs of SEs have wide fields of view and function as motion detectors [48].

### Homology of spider eyes with eyes of other arthropods

The last common ancestor of euarthropods is thought to have been equipped with a pair of lateral facetted eye and two pairs of median ocelli-type eyes. This eye ground pattern changed in the different lineages, yielding the great variability in visual equipment present today among euarthropods [47,49]. Arachnids have two externally visible PEs that are regarded as median eyes (reviewed [47]). Works of Homann suggest that the PEs are homologous to ocelli (reviewed by [47]). Onychophoran eyes show a mixture of annelid and arthropod characters [47], and a homology of the onychophoran eyes with the median or lateral ocelli of euarthropods has been suggested [50,51]. The consensus is thus that the median eyes of chelicerates, the crustacean nauplius eyes, and the insect ocelli are homologous. All these eye types demonstrate a *pax*-dependent development pathway that further supports their homology at the molecular level.

Spiders lack lateral facetted eyes but instead have up to three pairs of ocelli-type lateral eyes that are believed to have evolved from a facetted eye of the type present in *Limulus*. One suggestion is that the original facetted eye splits up into separate units, each with a common cuticular lens, a condition present among present day scorpions; this ommatidial organization was subsequently lost in the spider lineage. It is also possible that the lateral eyes of spiders evolved from three separate pairs of ommatidia [47]. The present study shows that each of the four eye pairs of spiders expresses a unique combination of RDGN genes. The separate RDGN profile of the spider eyes suggests another evolutionary scenario, namely a *de novo* origin of each of the lateral and/or median eyes in the arachnid lineage (except scorpions). If the lateral eyes had evolved from a single facetted eye, they would be expected to share a common molecular patterning mechanism. This is especially the case because, at least in *C. salei*, all lateral eyes seem to have the same function [48] and express the same set of opsins [11,52]. Nonetheless, further molecular characterisation of the different lateral eyes might reveal differences that are currently unrecognisable. Hence, we cannot rule out the possibility of common origin of the lateral eyes with subsequent differentiation of function accompanied by a gradual shift in the RDGN.

## Conclusions

Here, we report for the first time the duplication of several genes of the RDGN in an arachnid. The two *pax6* genes show homology to *ey* and *toy* and demonstrate divergent expression patterns. This differential expression pattern is also true for the remaining duplicated genes, namely *six1*, *six3*, *atn*, *otx*, and *dac*. The elements of the RDGN demonstrate expression patterns differentially in different eye vesicles. Moreover, PE development shows *pax6a* (*ey*) expression, suggesting *pax6* dependence, although secondary eyes develop independently of *pax6* genes and show differential expression of several RDGN genes. Comparing this with the other arthropods suggests that *pax6*-dependent median eye development is a ground pattern of eye development in this group and that the ocelli of insects, the median eyes of chelicerates, and nauplius eyes can be homologised. The expression pattern also makes it possible to distinguish between secondary eyes and principal eyes, but differences among the secondary eyes are probably due to functional divergence and genetic drift. The expression of the genes extends to the anlagen of ONs of the eyes, and different eye ONs show different combinations of the transcription factors. For example, ON anlagen of median eyes is developed with *pax6b* expression, but lateral eyes show a combination of different genes independent of *pax6*. Similarly, different combinations of the RDGN genes pattern the mushroom bodies and arcuate bodies, which are considered to be the third optic neuropil of secondary and principal eyes, respectively.

## Additional files

Additional file 1:
**Primer sequences used for amplification of the genes.**
Additional file 2:
**Anti-alpha-acetylated tubulin and nuclear staining in prosomal shield (A) and ventral closure (B) stages.** These are the first stages showing any sign of nerve fibre formation in the embryos. We therefore assume that optic neuropils are formed later during development when whole-mount antibody staining is no longer technically possible anymore because the cuticle inhibits antibody penetration. Additional file 3:
**Phylogenetic tree of bilaterian** ***pax6*** **genes based on the paired domain, the homeodomain, and the conserved regions in the linker between the two DNA binding domains.** Arthropod *pax6* genes do cluster in two groups of *eyeless*- and *toy*-like genes as previously shown by Callaerts *et al*. 2006. *Csa-pax6a* (black arrow) clusters together with *Pte-pax6.1* as the sister group to *ey* genes (the pink box) *Csa-pax6b* (black arrow) clearly clusters together with *toy*-like genes as the sister group to *Pte-Pax6.2* gene (the yellow box) with the exception of *Aga-ey*. Additional file 4:
**Phylogenetic tree of bilaterian** ***dachshund*** **genes.** *dac1* of vertebrates and *dac* of protostomes form a monophylum each (purple and yellow boxes, respectively). Additional file 5:
**Phylogenetic tree of bilaterian** ***bHLH*** **genes with** ***twist*** **as outgroup.** The tree shows a monophylum for *neurogenin* (*ngn*), *Target of pox neuron* (*TAP*), *Scleraxis* (*scx*), and *atonal* and *amos* genes (yellow box) with the exception of *Ceratitis capitata ngn1, Musca domestica scx,* and *Nasonia vitripennis dmd* which cluster together with *atonal* and *amos*. This is probably due to mislabeling of the genes in automated annotation methods. Chelicerates’ atonal forms a monophylum with *Cs-atha* as the sister group to *Pte-ath1*. These two form a sister group to *Limulus ath* and *Cs-athb* forms a sister group to *Pte-ath2*. The sister relationship between *atonal* and *amos* is not clear. Other *Cupiennius bHLH* genes (*Cs-bHLH a, b*, and *c*) cluster together with *atonal 8*. Additional file 6:
**Phylogenetic tree of bilaterian** ***otx*** **genes with** ***aristaless*** **(** ***arx*** **) as outgroup.** The two orthologs of *otx* in chelicerates (named *otxa* and *otxb* in *Cupiennius*) do not correspond to *otx1* and *otx2* of vertebrates showing that the duplication of the genes in chelicerates had happened independently from duplication of *otx* in vertebrates. Arthropods’ *otx* genes do not form a monophylum, and position of chelicerates’ two orthologs within the arthropods is unresolved. Additional file 7:
**Phylogenetic tree of bilaterian** ***six1/2, six3,*** **and** ***six4*** **genes based on the** ***six*** **protein-protein interaction domain and the homeodomain.** The tree is rooted with *six4. six1/2* and *six3* genes form monophyla (purple and yellow, respectively). Spider’s *six1a* and *b* group with *six1/2* monophylum forming a sister relationship with each other and *six3a* and *b* group with *six3* genes forming a sister relationship with each other.

## Abbreviations

af: anterior furrow
ALEs: anterior-lateral eyes
AMEs: anterior-median eyes
INPs: invaginating neural precursors
lf: lateral furrow
ls: lateral subdivision
ms: medial subdivision
ON: optic neuropil
PEs: principal eyes
PLEs: posterior-lateral eyes
PMEs: posterior-median eyes
ps: prosomal shield
RDGN: retinal determination gene network
