# Supplementary material for: Differential expression of retinal determination genes in the principal and secondary eyes of Cupiennius salei Keyserling (1877)
Source: EvoDevo. 2015 Apr 28;6:16. doi: 10.1186/s13227-015-0010-x (PMC4450993; doi:10.1186/s13227-015-0010-x)
Supplement: Additional file 2: — Anti-alpha-acetylated tubulin and nuclear staining in prosomal shield (A) and ventral closure (B) stages. These are the first stages showing any sign of nerve fibre formation in the embryos. We therefore assume that optic neuropils are formed later during development when whole-mount antibody staining is no longer technically possible anymore because the cuticle inhibits antibody penetration. [file 13227_2015_10_MOESM2_ESM.docx]

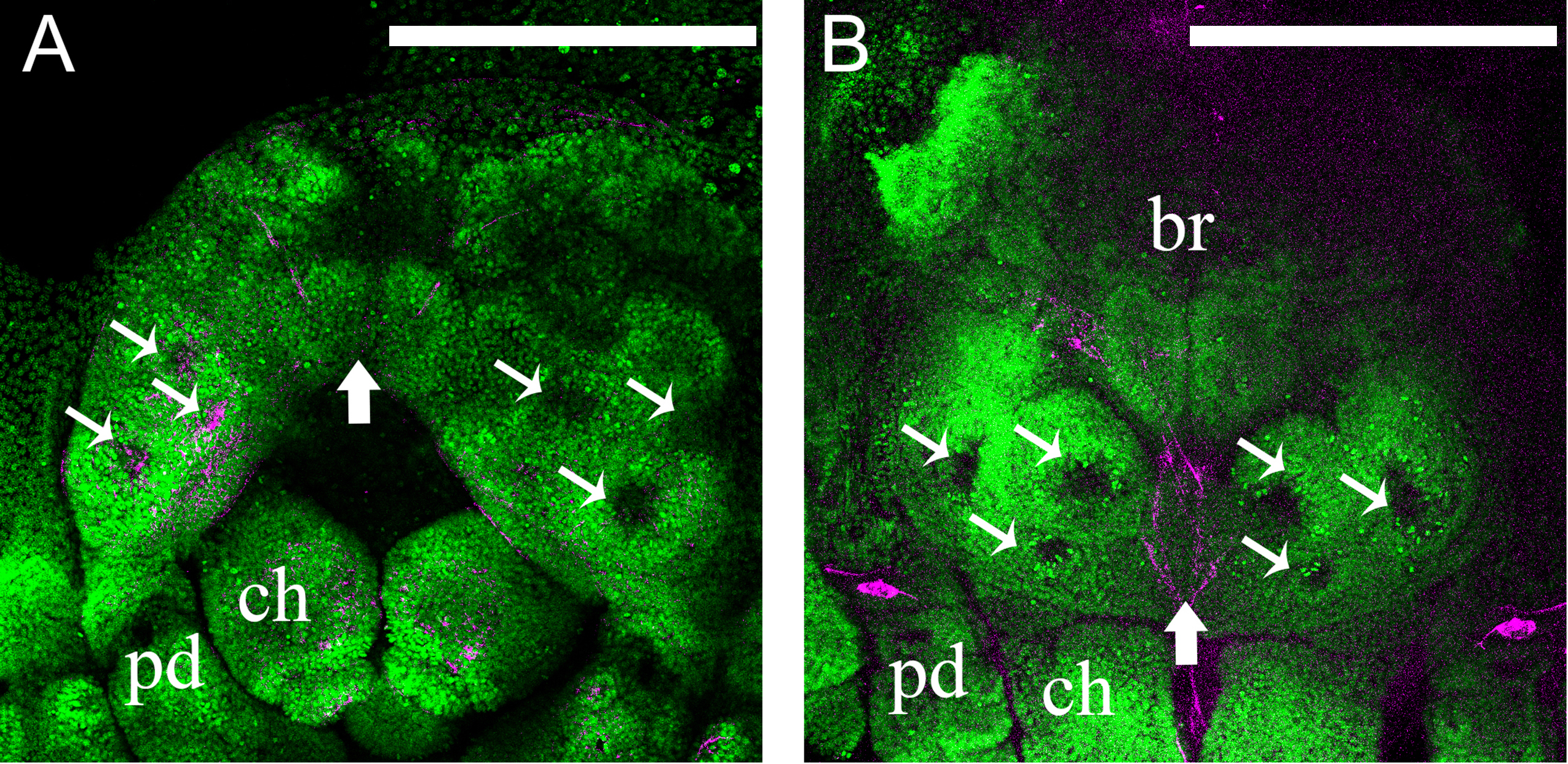


Anti-alpha-acetylated tubulin (magenta) and nuclear staining (green) in prosomal shield (A) and ventral closure (B) stages. These are the first stages showing any sign of nerve fibre formation in the embryos. We therefore assume that optic neuropils are formed later during development when whole-mount antibody staining is no longer technically possible anymore because the cuticle inhibits antibody penetration. Thin arrows: vesicles of secondary eyes, thick arrow: primary eyes. br: brain, ch: cheliceres, pd: pedipalps. Scale bar 200 μm.
