# Supplementary material for: Differential expression of retinal determination genes in the principal and secondary eyes of Cupiennius salei Keyserling (1877)
Source: EvoDevo. 2015 Apr 28;6:16. doi: 10.1186/s13227-015-0010-x (PMC4450993; doi:10.1186/s13227-015-0010-x)
Supplement: Additional file 3: — Phylogenetic tree of bilaterian pax6 genes based on the paired domain, the homeodomain, and the conserved regions in the linker between the two DNA binding domains. Arthropod pax6 genes do cluster in two groups of eyeless- and toy-like genes as previously shown by Callaerts et al. 2006. Csa-pax6a (black arrow) clusters together with Pte-pax6.1 as the sister group to ey genes (the pink box) Csa-pax6b (black arrow) clearly clusters together with toy-like genes as the sister group to Pte-Pax6.2 gene (the yellow box) with the exception of Aga-ey. [file 13227_2015_10_MOESM3_ESM.docx]

**
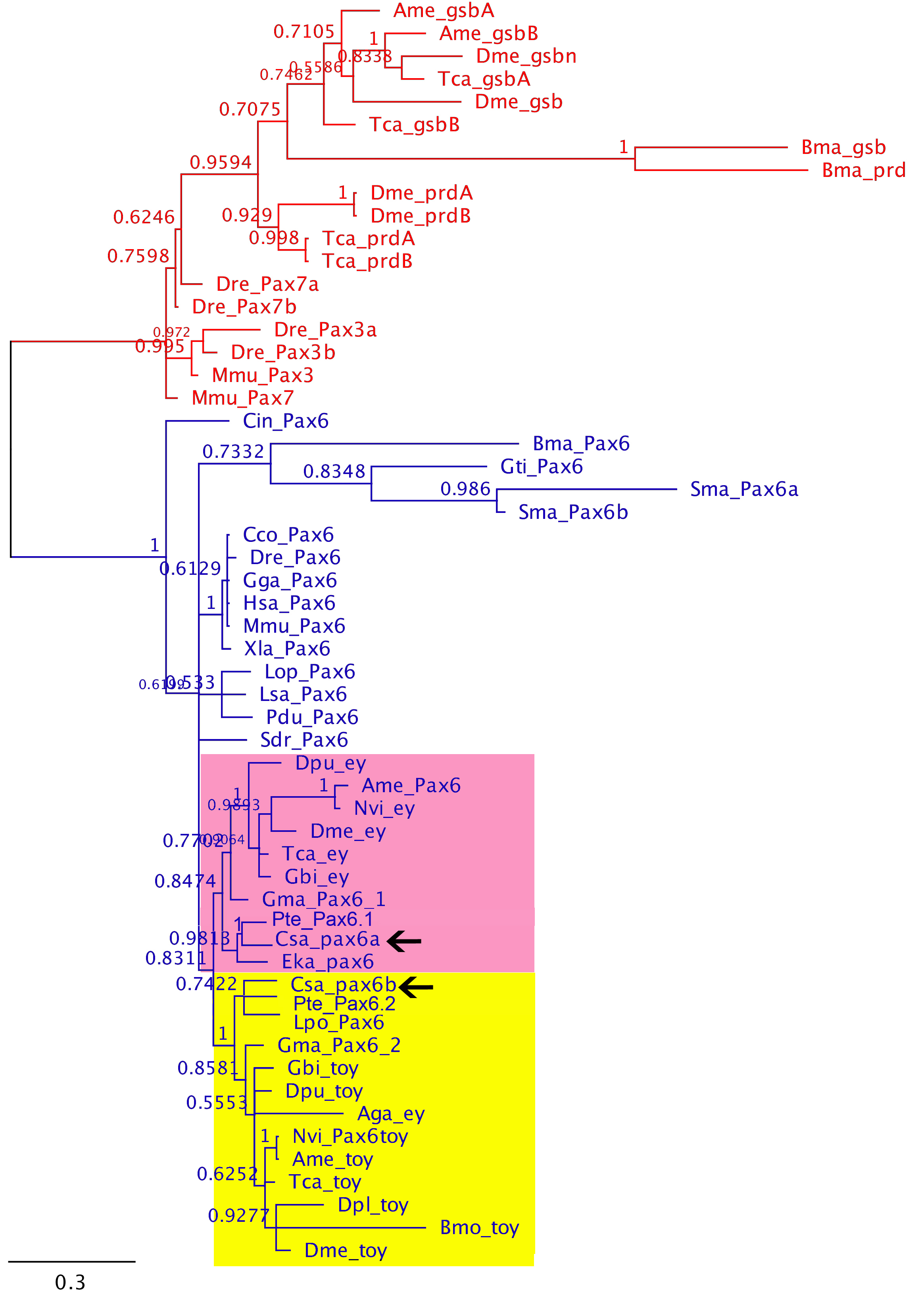
** Phylogenetic tree of bilaterian *pax6* genes based on the paired domain, the homeodomain, and the conserved regions in the linker between the two DNA binding domains. Bilaterian *pax6* and *pax3/7* protein sequences were obtained from the published literature or BLAST searches of the NCBI GenBank. The tree is built with the amino-acid sequences from Bayesian likelihood analysis using MrBayes with number of substitution types 6 and gamma rates, half compatibility consensus from two million replicates, burn-in of 10,000 replicates. Support values of branches are posterior probabilities of Bayesian likelihood. The resulted tree was viewed and handled by Treeview (v.1.6.5, http://taxonomy.zoology.gla.ac.uk/rod/rod.html). The tree is rooted with *pax3/7* genes as outgroup. Relevant here is the fact that arthropod *pax6* genes do cluster in two groups of *eyeless*- and *toy*-like genes as previously shown by Callaerts et al. 2006. *Csa-pax6a* (black arrow) clusters together with *Pte-pax6.1* as sister group to *ey* genes (the pink box) *Csa-pax6b* (black arrow) clearly clusters together with *toy*-like genes as sister group to *Pte-Pax6.2* gene (the yellow box) with the exception of *Aga-ey.*

**Table S2** List of the species names, phylum, and their abbreviation, the gene names and the GeneBank accession numbers used in phylogenetic analysis of *pax* genes.

| **Abbreviation** | **Accession info** | **Species name** | **Phylum** |
| --- | --- | --- | --- |
| Aga_ey | EAA06761 | *Anopheles gambiae* | Arthropoda |
| Ame_gsbA | XP_394847.3 | *Apis mellifera* | Arthropoda |
| Ame_gsbB | XP_394848 | *Apis mellifera* | Arthropoda |
| Ame_Pax6 | XP_006559917 | *Apis mellifera* | Arthropoda |
| Ame_toy | XP_006565439.1 | *Apis mellifera* | Arthropoda |
| Bfl_Pax6 | CAA11368 | Branchiostoma floridae | Chordata |
| Bma_Pax6  Bma_prd  Bma_gsb | XP_001899020.1  XP_001900160  XP_001900158.1 | *Brugia malayi* | Nematoda |
| Bmo_toy | NP_001189460 | *Bombyx mori* | Arthropoda |
| Cco_Pax6 | S37689 | *Coturnix coturnix* | Chordata |
| Cin_Pax6 | BAB85207 | *Ciona intestinalis* | Chordata |
| Csa_pax6a  Csa_Pax6b | LN624822  LN624823 | *Cupiennius salei* | Arthropoda |
| Dme_toy  Dmel_ey  Dme_prdA  Dme_prdB  Dme_gsb  Dme_gsbn | AAF59395.4  AAX52512.1  NP_523556.1  NP_723721.1  AAF47315  AAL49215 | *Drosophila melanogaster* | Arthropoda |
| Dpl_toy | EHJ78711 | *Danaus plexippus* | Arthropoda |
| Dre_Pax6  Dre_Pax3a  Dre_Pax3b  Dre_Pax7a  Dre_Pax7b | CAA44867  AAH76069  ACN88554  AAI63580  ACN88553 | *Danio rerio* | Chordata |
| Dpu_ey  Dpu_toy | EFX75780  EFX75784 | *Daphnia pulex* | Arthropoda |
| Eka_Pax6 | AGC51117 | *Euperipatoides kanangrensis* | Onychophora |
| *Gbi_ey*  *Gbi_toy* | BAM08281  BAM08280 | *Gryllus bimaculatus* | Arthropoda |
| Gga_Pax6 | NP990397 | *Gallus gallus* | Chordata |
| *Gma_Pax6.1*  *Gma_ Pax6.2* | CAE30301 CAE30302 | *Glomeris marginata* | Arthropoda |
| *Gti_ Pax6* | CAA09227 | *Girardia tigrina* | Platyhelminthes |
| *Hsa_ Pax6*  *Hsa_Pax3* | P26367  AAI14364.1 | *Homo sapiens* | Chordata |
| *Lsa_Pax6* | CAA64847 | *Lineus sanguineus* | Nemertea |
| *Lpo_Pax6* | EU673470 | *Limulus polyphemu* | Arthropoda |
| *Lop_Pax6* | AAB40616 | *Loligo opalescens* | Mollusca |
| *Mmu_Pax6*  *Mmu_Pax3*  *Mmu_Pax7* | P63015  AAH48699.1  NP_035169 | *Mus musculus* | Chordata |
| *Nvi-ey*  *Nvi_Pax6 toy* | ACT79977  ACT79981 | *Nasonia vitripennis* | Arthropoda |
| *Pdu_Pax6* | CAJ40659 | *Platynereis dumerilii* | Annelida |
| *Pte_Pax6.1* | M945394.1 | *Parasteatoda tepidariorum* | Arthropoda |
| *Pte_Pax6.2* | KP725068 | *Parasteatoda tepidariorum* | Arthropoda |
| *Sdr_Pax6* | ABB52751 | *Strongylocentrotus droebachiensis* | Echinodermata |
| *Sko Pax6* | AAP79294 | *Saccoglossus kowalevskii* | Hemichordata |
| *Sha_Pax6*  *Sma_Pax6* | KGB34115.1  CCD79874.1 | *Schistosoma haematobium*  *Schistosoma mansoni* | Platyhelminthes |
| *Tca_ey*  *Tca_Toy*  *Tca_prdA*  *Tca_prd B*  *Tca_gsbA*  *Tca_gsbB* | EFA02894 EFA02830 NP_001071090 EFA05752.1  XM_969092  XP_008197452.1  XP_008197453.1 | *Tribolium castaneum* | Arthropoda |
| *Xla_Pax6* | P55864 | *Xenopus laevis* | Chordata |
