# Supplementary material for: Differential expression of retinal determination genes in the principal and secondary eyes of Cupiennius salei Keyserling (1877)
Source: EvoDevo. 2015 Apr 28;6:16. doi: 10.1186/s13227-015-0010-x (PMC4450993; doi:10.1186/s13227-015-0010-x)
Supplement: Additional file 4: — Phylogenetic tree of bilaterian dachshund genes. dac1 of vertebrates and dac of protostomes form a monophylum each (purple and yellow boxes, respectively). [file 13227_2015_10_MOESM4_ESM.docx]

**
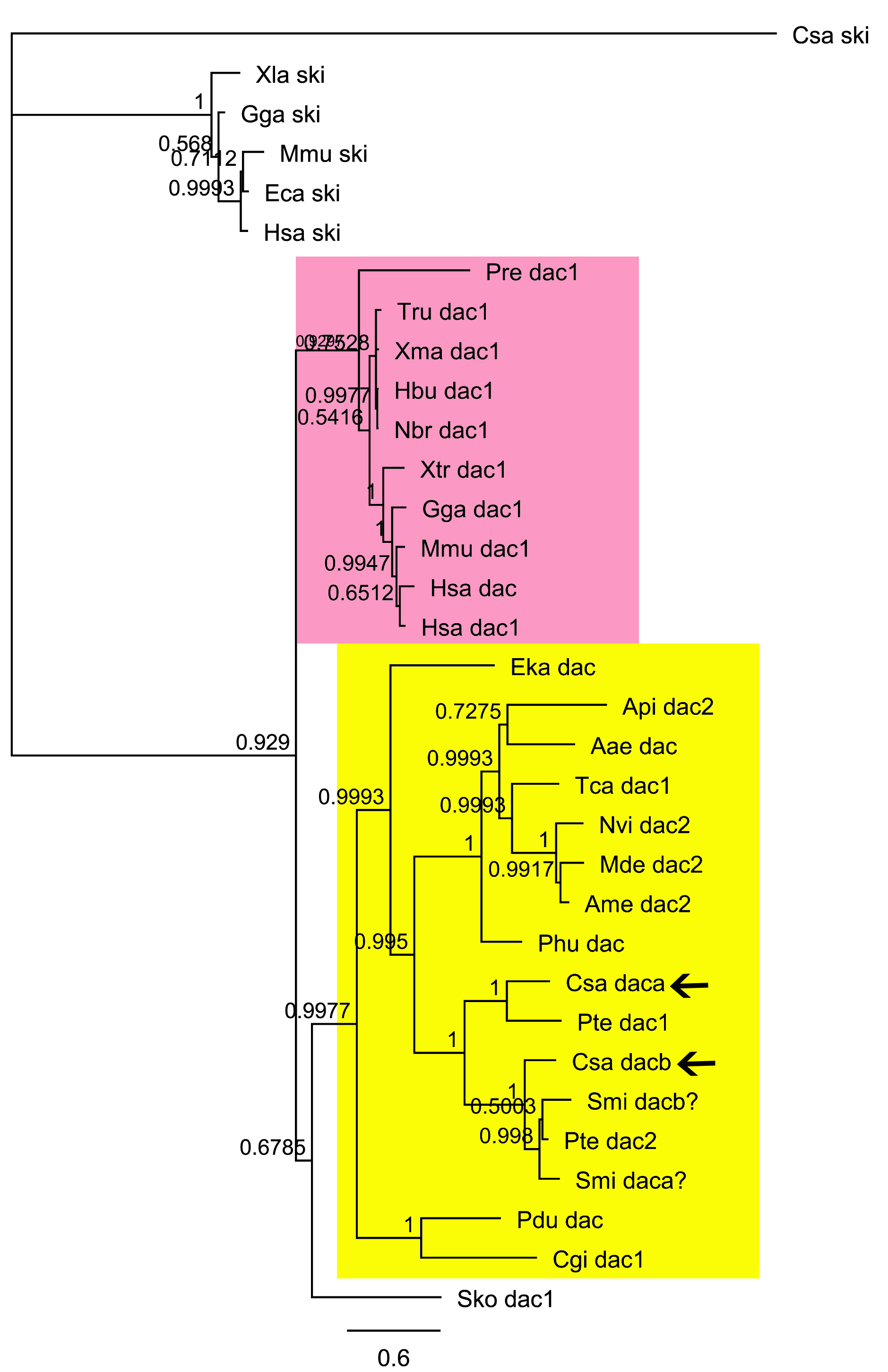
** Phylogenetic tree of bilaterian *dachshund* genes with *ski* as outgroup. Bilaterian *ski* and *dachshund* protein sequences were obtained from the published literature or BLAST searches of the NCBI GenBank. The tree is built with the amino-acid sequences from Bayesian likelihood analysis using MrBayes with number of substitution types 6 and gamma rates, half compatibility consensus from two million replicates, burn-in of 10,000 replicates. Support values of branches are posterior probabilities of Bayesian likelihood. *dac1* of vertebrates and *dac* of protostomes form a monophylum each (purple and yellow boxes respectively).

**Table S3** List of the species names, their phylum, and their abbreviation, the gene names and the GeneBank accession numbers used in phylogenetic analysis of *dachshund* genes.

| **Abbreviation** | **Accession info** | **Species name** | **Phylum** |
| --- | --- | --- | --- |
| Aae dac | XP_001662126 | *Aedes aegypti* | Arthropoda |
| Ame dac2 | XP_006567291 | *Apis mellifera* | Arthropoda |
| Api dac2 | XP_001945046 | *Acyrthosiphon pisum* | Arthropoda |
| Cgi dac1 | EKC27827 | *Crassostrea gigas* | Mollusca |
| Csa daca | LN624817 | *Cupiennius salei* | Arthropoda |
| Csa dacb | LN624818 | *Cupiennius salei* | Arthropoda |
| Csa ski | LN650634 | *Cupiennius salei* | Arthropoda |
| Eca ski | NP_001075287 | *Equus caballus* | Chordata |
| Eka dac | LN650632 | *Euperipatoides kanangrensis* | Arthropoda |
| Gga dac1 | NP_001186375 | *Gallus gallus* | Chordata |
| Gga ski | NP_001034407 | *Gallus gallus* | Chordata |
| Hbu dac1 | XP_005935211 | *Haplochromis burtoni* | Chordata |
| Hsa dac | NP_542937 | *Homo sapiens* | Chordata |
| Hsa ski | NP_003027 | *Homo sapiens* | Chordata |
| Mde dac2 | XP_008548052 | *Microplitis demolitor* | Arthropoda |
| Mmu dac1 | XP_006518572 | *Mus musculus* | Chordata |
| Mmu ski | AAA99669 | *Mus musculus* | Chordata |
| Nbr dac1 | XP_006804324 | *Neolamprologus brichardi* | Chordata |
| Nvi dac2 | XP_008204568 | *Nasonia vitripennis* | Arthropoda |
| Pdu dac | ADG26728 | *Platynereis dumerilii* | Annelida |
| Phu dac | XP_002427868 | *Pediculus humanus* | Arthropoda |
| Pre dac1 | XP_008427488 | *Poecilia reticulata* | Chordata |
| Pte dac1 | FM945397.1 | *Parasteatoda tepidariorum* | Arthropoda |
| Pte dac2 | KP725072 | *Parasteatoda tepidariorum* | Arthropoda |
| Sko dac1 | XP_006824867 | *Saccoglossus kowalevskii* | Hemichordata |
| Smi daca? | KFM61811 | *Stegodyphus mimosarum* | Arthropoda |
| Smi dacb? | KFM61812 | *Stegodyphus mimosarum* | Arthropoda |
| Tca dac1 | XP_008191786 | *Tribolium castaneum* | Arthropoda |
| Tru dac1 | XP_003961661 | *Takifugu rubripes* | Chordata |
| Xla dac1 | NP_001080930 | *Xenopus laevis* | Chordata |
| Xla ski | NP_001090187 | *Xenopus laevis* | Chordata |
| Xma dac1 | XP_005798076 | *Xiphophorus maculatus* | Chordata |
