# Supplementary material for: Differential expression of retinal determination genes in the principal and secondary eyes of Cupiennius salei Keyserling (1877)
Source: EvoDevo. 2015 Apr 28;6:16. doi: 10.1186/s13227-015-0010-x (PMC4450993; doi:10.1186/s13227-015-0010-x)
Supplement: Additional file 5: — Phylogenetic tree of bilaterian bHLH genes with twist as outgroup. The tree shows a monophylum for neurogenin (ngn), Target of pox neuron (TAP), Scleraxis (scx), and atonal and amos genes (yellow box) with the exception of Ceratitis capitata ngn1, Musca domestica scx, and Nasonia vitripennis dmd which cluster together with atonal and amos. This is probably due to mislabeling of the genes in automated annotation methods. Chelicerates’ atonal forms a monophylum with Cs-atha as the sister group to Pte-ath1. These two form a sister group to Limulus ath and Cs-athb forms a sister group to Pte-ath2. The sister relationship between atonal and amos is not clear. Other Cupiennius bHLH genes (Cs-bHLH a, b, and c) cluster together with atonal 8. [file 13227_2015_10_MOESM5_ESM.docx]

**
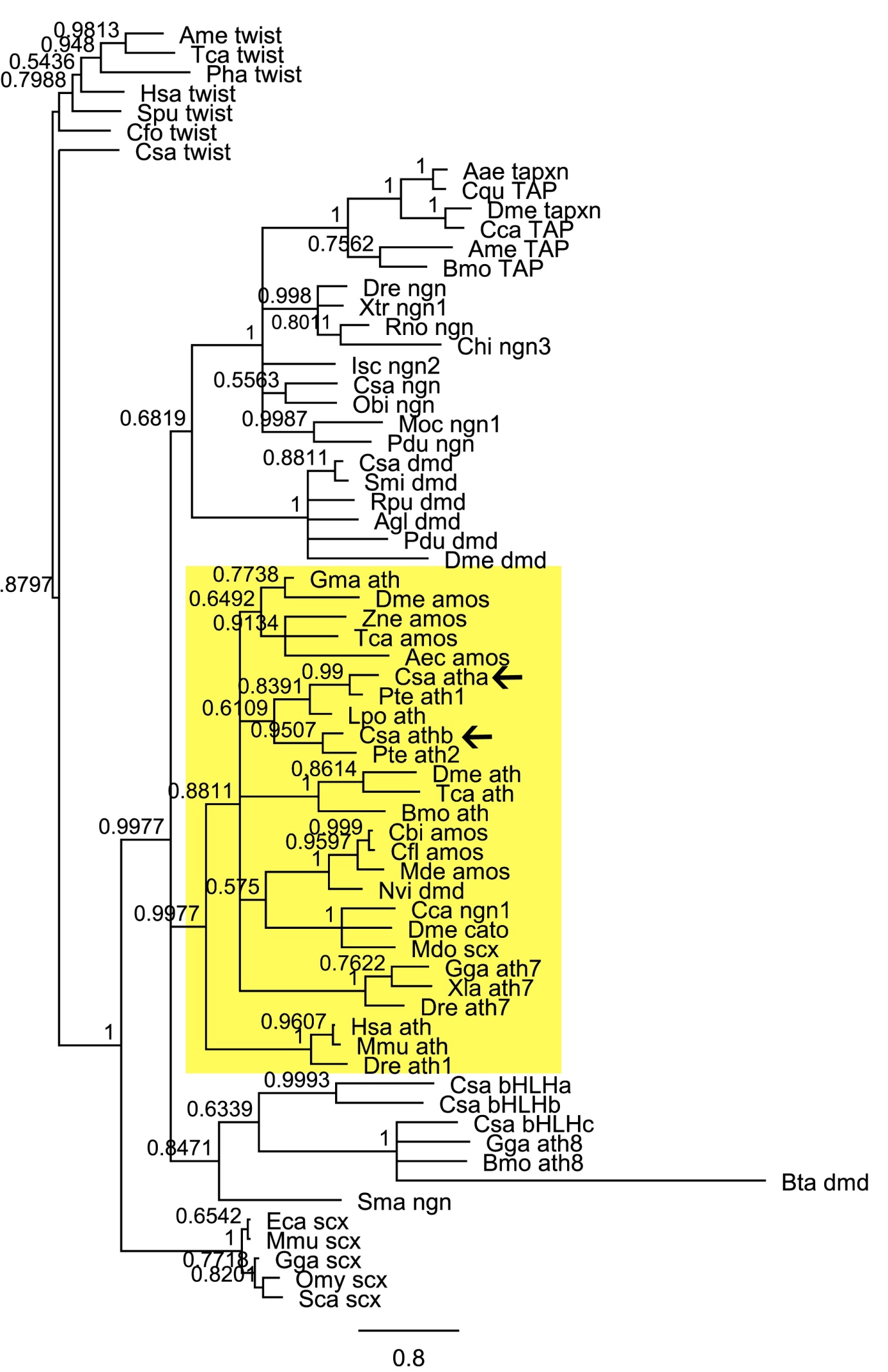
**Phylogenetic tree of bilaterian *bHLH* genes with *twist* as outgroup. Bilaterian*bHLH* protein sequences were obtained from the published literature or BLAST searches of the NCBI GenBank. The tree is built with the amino-acid sequences from Bayesian likelihood analysis using MrBayes with number of substitution types 6 and gamma rates, half compatibility consensus from two million replicates, burn-in of 10,000 replicates. Support values of branches are posterior probabilities of Bayesian likelihood. The tree shows a monophylum for *neurogenin* (*ngn*), *Target of pox neuron* (*TAP*), *Scleraxis* (*scx*), and *atonal* and *amos* genes (yellow box) with the exception of *Ceratitis capitata ngn1, Musca domestica scx,* and *Nasonia vitripennis dmd* which cluster together with *atonal* and *amos*. This is probably due to mislabeling of the genes in automated annotation methods. Chelicerates’ atonal forms a monophylum with *Cs-atha* as sister group to *Pte-ath1*. These two form a sister group to *Limulus* *ath* and *Cs-athb* forms a sister group to *Pte-ath2*. The sister relationship between *atonal* and *amos* is not clear. Other *Cupiennius* *bHLH* genes (*Cs-bHLH a, b*, and *c*) cluster together with *atonal 8*.

**Table S3** List of the species names, phylum, and their abbreviation, the gene names and the GeneBank accession numbers used in phylogenetic analysis of *bHLH* genes.

| **Abbreviation** | **Accession info** | **Species name** | **Phylum** |
| --- | --- | --- | --- |
| Aec amos | [GL888577.1](http://www.ncbi.nlm.nih.gov/nuccore/332018829) | Acromyrmex echinatior | Arthropoda |
| Agl dmd | [GALX01001891.1](http://www.ncbi.nlm.nih.gov/nuccore/550259434) | *Anoplophora glabripennis* | Arthropoda |
| Ame TAP | [XM_006562259.1](http://www.ncbi.nlm.nih.gov/nuccore/571564375) | *Apis mellifera* | Arthropoda |
| Ame twist | XP_396700 | *Apis mellifera* | Arthropoda |
| Bmo ath | NP_001243976.1 | *Bombyx mori* | Arthropoda |
| Bmo ath8 | NP_001268821.1 | *Bombyx mori* | Arthropoda |
| Bmo TAP | XP_004927170 | *Bombyx mori* | Arthropoda |
| Bta dmd | XP_002692729 | *Bos taurus* | Chordata |
| Cbi amos | EZA47885.1 | *Cerapachys biroi* | Arthropoda |
| Cca ngn1 | JAC03899 | *Ceratitis capitata* | Arthropoda |
| Cca TAP | XP_004530894 | *Ceratitis capitata* | Arthropoda |
| Cfl amos | EFN72435 | *Camponotus floridanus* | Arthropoda |
| Cfo twist | ADI48177 | *Crepidula fornicata* | Arthropoda |
| Chi ngn3 | AGF25286 | *Capra hircus* | Chordata |
| Cqu TAP | EDS34226 | *Culex quinquefasciatus* | Arthropoda |
| Csa atha | LN624815 | *Cupiennius salei* | Arthropoda |
| Csa athb | LN624816 | *Cupiennius salei* | Arthropoda |
| Csa bHLHa, fer3 | LN650635 | *Cupiennius salei* | Arthropoda |
| Csa bHLHb | LN650636 | *Cupiennius salei* | Arthropoda |
| Csa bHLHc, ath8 | LN650637 | *Cupiennius salei* | Arthropoda |
| Csa dmd | LN650638 | *Cupiennius salei* | Arthropoda |
| Csa ngn | LN650639 | *Cupiennius salei* | Arthropoda |
| Csa twist | LN650640 | *Cupiennius salei* | Arthropoda |
| Dme amos | NP_477446 | *Drosophila melanogaster* | Arthropoda |
| Dme ath | NP_731223 | *Drosophila melanogaster* | Arthropoda |
| Dme cato | NP_477344 | *Drosophila melanogaster* | Arthropoda |
| Dme dmd | NP_523611 | *Drosophila melanogaster* | Arthropoda |
| Dme tapxn | NP_524124 | *Drosophila melanogaster* | Arthropoda |
| Dre ath1 | NP_571166 | *Danio rerio* | Chordata |
| Dre ath7 | NP_571707 | *Danio rerio* | Chordata |
| Dre ngn | NP_571116 | *Danio rerio* | Chordata |
| Eca scx | NP_001098620 | *Equus caballus* | Chordata |
| Gga ath7 | NP_989999 | *Gallus gallus* | Chordata |
| Gga ath8 | NP_001171210 | *Gallus gallus* | Chordata |
| Gga scx | NP_989584 | *Gallus gallus* | Chordata |
| Gma ath | ABF21051 | *Glomeris marginata* | Arthropoda |
| Hsa ath | NP_005163 | *Homo sapiens* | Chordata |
| Hsa twist | XP_006715832 | *Homo sapiens* | Chordata |
| Isc ngn2 | XP_002409093 | *Ixodes scapularis* | Arthropoda |
| Lpo ath | ACD88753 | *Limulus polyphemus* | Arthropoda |
| Mde amos | XP_008549313 | *Microplitis demolitor* | Arthropoda |
| Mdo scx | XP_005189324 | *Musca domestica* | Arthropoda |
| Mmu ath | NP_031526 | *Mus musculus* | Chordata |
| Mmu scx | NP_942588 | *Mus musculus* | Chordata |
| Moc ngn1 | XP_003740423 | Metaseiulus occidentalis |  |
| Nvi dmd | XP_008204411 | *Nasonia vitripennis* | Arthropoda |
| Obi ngn | ADK23852 | *Octopus bimaculoides* | Mollusca |
| Omy scx | AHM24917 | *Oncorhynchus mykiss* | Chordata |
| Pdu dmd | AFV92894 | *Platynereis dumerilii* | Annelida |
| Pdu ngn | CAQ57535 | *Platynereis dumerilii* | Annelida |
| Pha twist | ABG89389 | *Parhyale hawaiensis* | Arthropoda |
| Pte ath1 |  |  | Arthropoda |
| Pte ath2 |  |  | Arthropoda |
| Rno ngn | NP_062080 | *Rattus norvegicus* | Chordata |
| Rpu dmd | JAA56849 | *Rhipicephalus pulchellus* | Arthropoda |
| Sca scx | ABI34478 | *Scyliorhinus canicula* | Chordata |
| Sma ngn | CCD75861 | *Schistosoma mansoni* | Platyhelminthes |
| Smi dmd | KFM72997 | *Stegodyphus mimosarum* | Arthropoda |
| Spu twist | NP_001099179 | *Strongylocentrotus purpuratus* | Echinodermata |
| Tca amos | XP_974297 | *Tribolium castaneum* | Arthropoda |
| Tca ath | XP_970709 | *Tribolium castaneum* | Arthropoda |
| Tca twist | NP_001034496 | *Tribolium castaneum* | Arthropoda |
| Xla ath7 | NP_001079290 | *Xenopus laevis* | Chordata |
| Xtr ngn1 | NP_001116895 | *Xenopus(Silurana) tropicalis* | Chordata |
| Zne amos | KDR11020 | *Zootermopsis nevadensis* | Arthropoda |
