# Supplementary material for: Differential expression of retinal determination genes in the principal and secondary eyes of Cupiennius salei Keyserling (1877)
Source: EvoDevo. 2015 Apr 28;6:16. doi: 10.1186/s13227-015-0010-x (PMC4450993; doi:10.1186/s13227-015-0010-x)
Supplement: Additional file 6: — Phylogenetic tree of bilaterian otx genes with aristaless ( arx ) as outgroup. The two orthologs of otx in chelicerates (named otxa and otxb in Cupiennius) do not correspond to otx1 and otx2 of vertebrates showing that the duplication of the genes in chelicerates had happened independently from duplication of otx in vertebrates. Arthropods’ otx genes do not form a monophylum, and position of chelicerates’ two orthologs within the arthropods is unresolved. [file 13227_2015_10_MOESM6_ESM.docx]

**
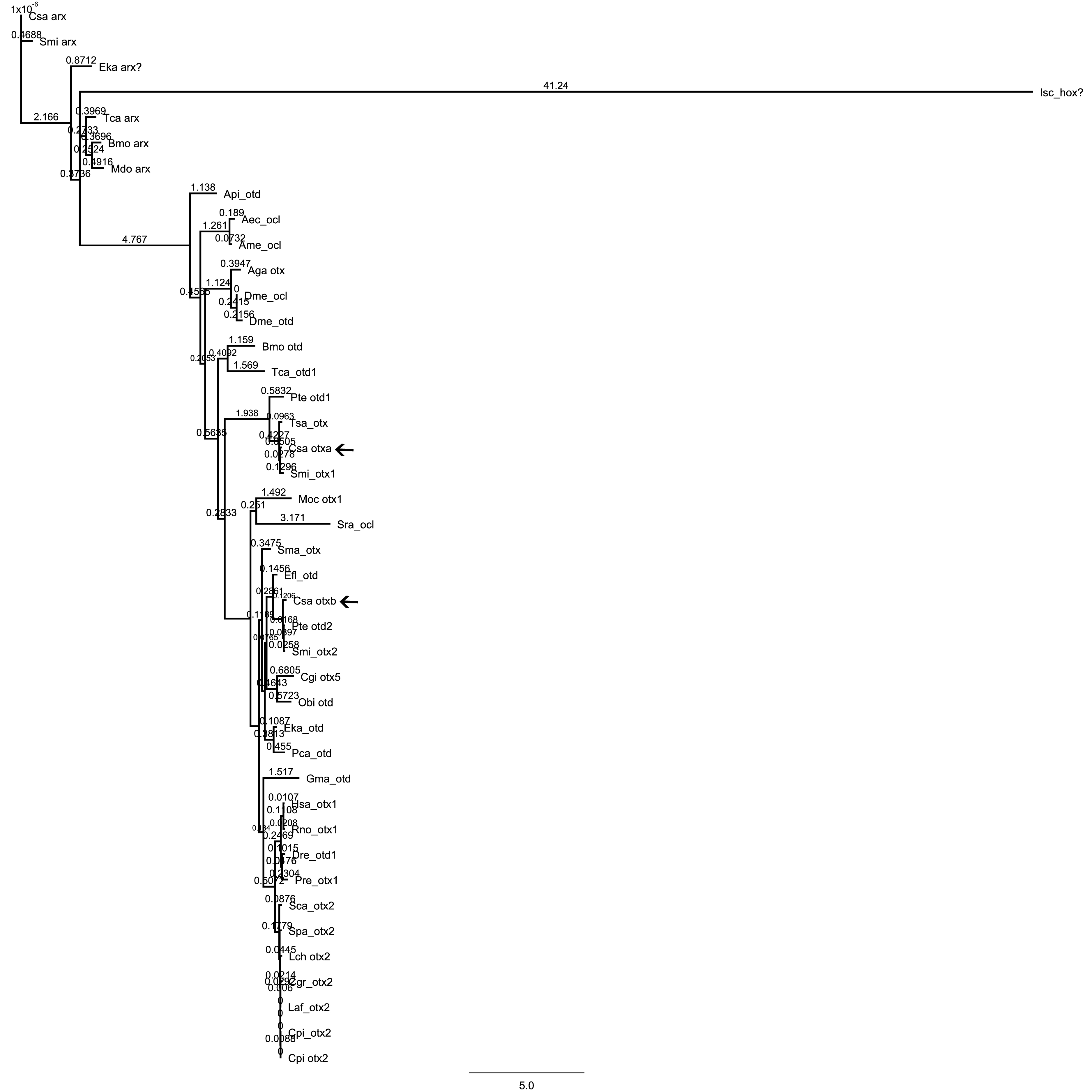
**Phylogenetic tree of bilaterian *otx* genes with *aristaless* (*arx*) as outgroup. Protein sequences were obtained from the published literature or BLAST searches of the NCBI GenBank. The tree is built with the amino-acid sequences from Maximum likelihood analysis. The two orthologs of *otx* in chelicerates (named *otxa* and *otxb* in *Cupiennius*) does not correspond to *otx1* and *otx2* of vertebrates showing that the duplication of the genes in chelicerates had happened independently from duplication of *otx* in vertebrates. Arthropods’ *otx* genes do not form a monophylum and position of chelicerates’ two orthologs within the arthropods is unresolved.

**Table S5** List of the species names, their phylum, and their abbreviation, the gene names and the GeneBank accession numbers used in phylogenetic analysis of *otx* genes.

| **Abbreviation** | **Accession info** | **Species name** | **Phylum** |
| --- | --- | --- | --- |
| Aec_ocl | EGI70731 | *Acromyrmex echinatior* | Arthropoda |
| Aga_otx | XP_310918 | *Anopheles gambiae* | Arthropoda |
| Ame_ocl | XP_006571327 | *Apis mellifera* | Arthropoda |
| Api_otd | XP_001948393 | *Acyrthosiphon pisum* | Arthropoda |
| Bmo_arx | XP_004929200 | *Bombyx mori* | Arthropoda |
| Bmo_otd | XP_310918 | *Bombyx mori* | Arthropoda |
| Cgi otx5 | EKC28141 | *Crassostrea gigas* | Mollusca |
| Cgr_otx2 | ERE92634 | *Cricetulus griseus* | Chordata |
| Cpi otx2 | XP_005295567 | *Chrysemys picta* | Chordata |
| Csa otxa | LN624820 | *Cupiennius salei* | Arthropoda |
| Csa otxb | LN624821 | *Cupiennius salei* | Arthropoda |
| Csa_arx | To be submitted | *Cupiennius salei* | Arthropoda |
| Dme_ocl | NP_001014727 | *Drosophila melanogaster* | Arthropoda |
| Dme_otd | CAA41732 | *Drosophila melanogaster* | Arthropoda |
| Dre_otd1 | NP_571325 | *Danio rerio* | Chordata |
| Efl_otd | AAU85255 | *Euscorpius flavicaudis* | Arthropoda |
| Eka arx | CDK60408 | *Euperipatoides kanangrensis* | Onychophora |
| Eka_otd | LN650633 | *Euperipatoides kanangrensis* | Onychophora |
| Gma_otd | CAK50843 | *Glomeris marginata* | Arthropoda |
| Hsa_otx1 | NP_055377 | *Homo sapiens* | Chordata |
| Isc_hox? | XP_002403318 | *Ixodes scapularis* | Arthropoda |
| Laf_otx2 | XP_003408715 | *Loxodonta africana* | Chordata |
| Lch otx2 | XP_005986517 | *Latimeria chalumnae* | Chordata |
| Mdo_arx | XP_005181478 | *Musca domestica* | Arthropoda |
| Moc_otx1 | XP_003742052 | *Metaseiulus occidentalis* | Arthropoda |
| Obi_otd | AAZ99218 | *Octopus bimaculoides* | Mollusca |
| Pca_otd | AFY12008 | *Priapulus caudatus* | Priapulida |
| Pre_otx1 | XP_008416164 | *Poecilia reticulata* | Chordata |
| Pte otd1 | AB096074.1 | *Parasteatoda tepidariorum* | Arthropoda |
| Pte otd2 | KP725073 | *Parasteatoda tepidariorum* | Arthropoda |
| Rno_otx1 | NP_037241 | *Rattus norvegicus* | Chordata |
| Sca_otx2 | AAP04272 | *Scyliorhinus canicula* | Chordata |
| Sma_otx | ABY74501 | *Strigamia maritima* | Arthropoda |
| Smi_arx | KFM57110 | *Stegodyphus mimosarum* | Arthropoda |
| Smi_otx1 | KFM58880 | *Stegodyphus mimosarum* | Arthropoda |
| Smi_otx2 | KFM60429 | *Stegodyphus mimosarum* | Arthropoda |
| Spa_otx2 | XP_008281544 | *Stegastes partitus* | Chordata |
| Sra_ocl | CEF62108 | *Strongyloides ratti* | Nematoda |
| Tca_otd1 | NP_001034513 | *Tribolium castaneum* | Arthropoda |
| Tca_arx | XP_008193910 | *Tribolium castaneum* | Arthropoda |
| Tsa_otx | AAU85252 | *Tegenaria saeva* | Arthropoda |
