# Supplementary material for: Differential expression of retinal determination genes in the principal and secondary eyes of Cupiennius salei Keyserling (1877)
Source: EvoDevo. 2015 Apr 28;6:16. doi: 10.1186/s13227-015-0010-x (PMC4450993; doi:10.1186/s13227-015-0010-x)
Supplement: Additional file 7: — Phylogenetic tree of bilaterian six1/2, six3, and six4 genes based on the six protein-protein interaction domain and the homeodomain. The tree is rooted with six4. six1/2 and six3 genes form monophyla (purple and yellow, respectively). Spider’s six1a and b group with six1/2 monophylum forming a sister relationship with each other and six3a and b group with six3 genes forming a sister relationship with each other. [file 13227_2015_10_MOESM7_ESM.docx]

**
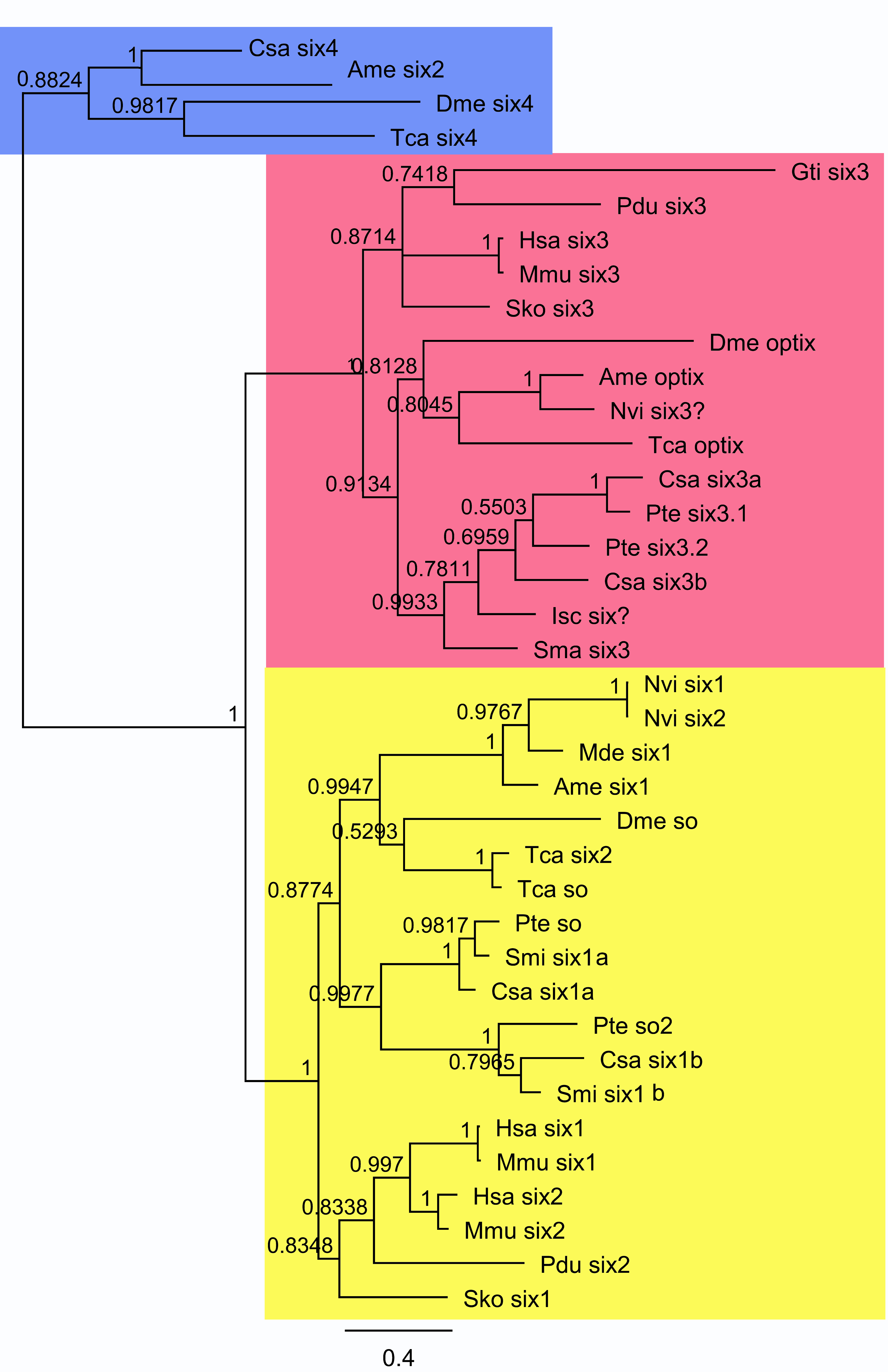
** Phylogenetic tree of bilaterian *six1/2, six3,* and *six4* genes based on the *six* protein-protein interaction domain and the homeodomain. Bilaterian *six* protein sequences were obtained from the published literature or BLAST searches of the NCBI GenBank. The tree is built with the amino-acid sequences from Bayesian likelihood analysis using MrBayes with number of substitution types 6 and gamma rates, half compatibility consensus from two million replicates, burn-in of 10,000 replicates. Support values of branches are posterior probabilities of Bayesian likelihood. The tree is rooted with *six4*. *six1/2* and *six3* genes form monophyla (purple and yellow, respectively). Spider’s *six1a* and *b* group with six1/2 monophylum and form sister relationship with each other and *six3a* and *b* group with *six3* genes forming sister relationship with each other.

**Table S6** List of the species names, their phylum, and their abbreviation, the gene names and the GeneBank accession numbers used in phylogenetic analysis of *six* genes.

| **Abbreviation** | **Accession info** | **Species name** | **Phylum** |
| --- | --- | --- | --- |
| Ame optix | XP_623764.2 | *Apis mellifera* | Arthropoda |
| Ame six1 | XP_006564253.1 | *Apis mellifera* | Arthropoda |
| Ame six2 | [XP_001120698.2](http://www.ncbi.nlm.nih.gov/protein/328791573?report=genbank&log$=prottop&blast_rank=1&RID=4A51YNFE015) | *Apis mellifera* | Arthropoda |
| Csa six1a | LN624824 | *Cupiennius salei* | Arthropoda |
| Csa six1b | LN624825 | *Cupiennius salei* | Arthropoda |
| Csa six3a | LN624826 | *Cupiennius salei* | Arthropoda |
| Csa six3b | LN624827 | *Cupiennius salei* | Arthropoda |
| Csa six4 | LN624828 | *Cupiennius salei* | Arthropoda |
| Dme optix | AAF59147 | *Drosophila melanogaster* | Arthropoda |
| Dme six4 | [NP_649256.1](http://www.ncbi.nlm.nih.gov/protein/21356333?report=genbank&log$=prottop&blast_rank=1&RID=4A59M4HD014) | *Drosophila melanogaster* | Arthropoda |
| Dme so | AAF59260 | *Drosophila melanogaster* | Arthropoda |
| Gti six3 | AAN77127 | *Girardia tigrina* | Platyhelminthes |
| Hsa six1 | AAK06772 | *Homo sapiens* | Chordata |
| Hsa six2 | AAK06773 | *Homo sapiens* | Chordata |
| Hsa six3 | AAD15753 | *Homo sapiens* | Chordata |
| Isc six3 | XP_002405217.1 | *Ixodes scapularis* | Arthropoda |
| Mde six1 | XP_008550384 | *Microplitis demolitor* | Arthropoda |
| Mmu six1 | [NP_033215.2](http://www.ncbi.nlm.nih.gov/protein/226958387?report=genbank&log$=prottop&blast_rank=1&RID=4A5D2Y3B014) | *Mus musculus* | Chordata |
| Mmu six2 | BAA11825 | *Mus musculus* | Chordata |
| Mmu six3 | [NP_035511.2](http://www.ncbi.nlm.nih.gov/protein/59939908?report=genbank&log$=prottop&blast_rank=1&RID=4A5TCY5F015) | *Mus musculus* | Chordata |
| Nvi six1 | XP_001600428 | *Nasonia vitripennis* | Arthropoda |
| Nvi six2 | XP_008211209 | *Nasonia vitripennis* | Arthropoda |
| Nvi six3? | XP_008217298 | *Nasonia vitripennis* | Arthropoda |
| Pdu six2 | CAC86663 | *Platynereis dumerilii* | Annelida |
| Pdu six3 | [CAR66435.1](http://www.ncbi.nlm.nih.gov/protein/202957446?report=genbank&log$=prottop&blast_rank=1&RID=4A5WPVJH01R) | *Platynereis dumerilii* | Annelida |
| Pte six3_1 | [BAK93300.1](http://www.ncbi.nlm.nih.gov/protein/348600221?report=genbank&log$=prottop&blast_rank=1&RID=4A6X4C20014) | *Parasteatoda tepidariorum* | Arthropoda |
| Pte six3_2 | [BAK93301.1](http://www.ncbi.nlm.nih.gov/protein/348600223?report=genbank&log$=prottop&blast_rank=2&RID=4A6X4C20014) | *Parasteatoda tepidariorum* | Arthropoda |
| Pte so1 | KP725069 | *Parasteatoda tepidariorum* | Arthropoda |
| Pte so2 | KP725070 | *Parasteatoda tepidariorum* | Arthropoda |
| Sko six1 | [NP_001277017.1](http://www.ncbi.nlm.nih.gov/protein/586946361?report=genbank&log$=prottop&blast_rank=1&RID=4A720K02014) | *Saccoglossus kowalevskii* | Hemichordata |
| Sko six3 | AAP79281 | *Saccoglossus kowalevskii* | Hemichordata |
| Sma six3 | ABY74502 | *Strigamia maritima* | Arthropoda |
| Smi six1a | KFM62344 | *Stegodyphus mimosarum* | Arthropoda |
| Smi six1b | KFM77431 | *Stegodyphus mimosarum* | Arthropoda |
| Tca optix | CAP58434 | *Tribolium castaneum* | Arthropoda |
| Tca six2 | XP_008192976 | *Tribolium castaneum* | Arthropoda |
| Tca six4 | [XP_008191364.1](http://www.ncbi.nlm.nih.gov/protein/642917868?report=genbank&log$=prottop&blast_rank=1&RID=4A7G2PF601R) | *Tribolium castaneum* | Arthropoda |
| Tca so | ACS50141 | *Tribolium castaneum* | Arthropoda |
